# CPEB2-activated *Prdm16* translation promotes brown adipocyte function and prevents obesity

**DOI:** 10.1016/j.molmet.2024.102034

**Published:** 2024-09-19

**Authors:** Wen-Hsin Lu, Hui-Feng Chen, Pei-Chih King, Chi Peng, Yi-Shuian Huang

**Affiliations:** Institute of Biomedical Sciences, Academia Sinica, Taipei 11529, Taiwan

**Keywords:** Brown adipose tissue, CPEB2, Obesity, PRDM16, Thermogenesis, Translational control

## Abstract

**Objective:**

Brown adipose tissue (BAT) plays an important role in mammalian thermogenesis through the expression of uncoupling protein 1 (UCP1). Our previous study identified cytoplasmic polyadenylation element binding protein 2 (CPEB2) as a key regulator that activates the translation of *Ucp1* with a long 3′-untranslated region (*Ucp1L*) in response to adrenergic signaling. Mice lacking CPEB2 or *Ucp1L* exhibited reduced UCP1 expression and impaired thermogenesis; however, only CPEB2-null mice displayed obesogenic phenotypes. Hence, this study aims to investigate how CPEB2-controlled translation impacts body weight.

**Methods:**

Body weight measurements were conducted on mice with global knockout (KO) of CPEB2, UCP1 or *Ucp1L*, as well as those with conditional knockout of CPEB2 in neurons or adipose tissues. RNA sequencing coupled with bioinformatics analysis was used to identify dysregulated gene expression in CPEB2-deficient BAT. The role of CPEB2 in regulating PRD1-BF1-RIZ1 homologous-domain containing 16 (PRDM16) expression was subsequently confirmed by RT-qPCR, Western blotting, polysomal profiling and luciferase reporter assays. Adeno-associated viruses (AAV) expressing CPEB2 or PRDM16 were delivered into BAT to assess their efficacy in mitigating weight gain in CPEB2-KO mice.

**Results:**

We validated that defective BAT function contributed to the increased weight gain in CPEB2-KO mice. Transcriptomic profiling revealed upregulated expression of genes associated with muscle development in CPEB2-KO BAT. Given that both brown adipocytes and myocytes stem from myogenic factor 5-expressing precursors, with their cell-fate differentiation regulated by PRDM16, we identified that *Prdm16* was translationally upregulated by CPEB2. Ectopic expression of PRDM16 in CPEB2-deprived BAT restored gene expression profiles and decreased weight gain in CPEB2-KO mice.

**Conclusions:**

In addition to *Ucp1L*, activation of *Prdm16* translation by CPEB2 is critical for sustaining brown adipocyte function. These findings unveil a new layer of post-transcriptional regulation governed by CPEB2, fine-tuning thermogenic and metabolic activities of brown adipocytes to control body weight.

## Highlights

• CPEB2-KO mice exhibit increased weight gain and body fat accumulation.
• Mice lacking CPEB2 in adipose tissues show elevated weight gain.
• Upregulated myogenic gene expression in CPEB2-deficient BAT.
• Ectopic expression of CPEB2 or PRDM16 in BAT reduces weigh gain in CPEB2-KO mice.
• CPEB2-activated *Prdm16* translation suppresses myogenic remodeling and prevent obesit.

## 1 Introduction

Obesity, caused by accumulation of excess energy over time, has emerged as a major epidemic in many industrialized countries and strongly affected public health due to its strong association with cardiovascular disease and type 2 diabetes mellitus [1,2]. Energy intake is expended by physical work and metabolic heat generation, making individuals with reduced metabolic activity more prone to weight gain. Brown adipose tissue (BAT) dissipates chemical energy in the form of heat through the action of uncoupling protein 1 (UCP1) in the inner mitochondrial membrane [3,4]. Studies using positron-emission tomography to monitor ^18^F-fluorodeoxyglucose (^18^F-FDG) uptake have identified BAT activity in adult humans [5,6], showing a positive correlation with resting metabolic rate while inversely related to body mass index (BMI) and percentage body fat [6]. Despite the hereditary component of obesity susceptibility, lifestyle and dietary habits also exert substantial influence on its development, rendering the identification of genetic predispositions to weight gain and obesity challenging.

Cytoplasmic polyadenylation element binding protein 2 (CPEB2), a sequence-specific RNA-binding protein, regulates target mRNA translation for various physiological functions [7,8], including neonatal respiration, memory consolidation, pulmonary alveologenesis and mammary gland development [[9], [10], [11], [12]]. Moreover, CPEB2 is pivotal for sustaining thermogenic and metabolic activities of BAT. Previously, we identified two distinct *Ucp1* transcripts varying in the 3′-untranslated region (3′-UTR), which undergo differential translation in response to β3-adrenergic receptor (β3AR) signaling [13,14]. Approximately 10% of mouse *Ucp1* mRNA has a long 3′-UTR (referred to as *Ucp1L*), which is translationally activated by CPEB2 to supply half of the UCP1 in BAT. Dysregulated expression of UCP1 in beige or brown adipocytes often correlates with lean or obese phenotypes in genetically modified mice [[15], [16], [17], [18]]; however, UCP1-KO mice exhibit high resistance to both diet-induced obesity and genetic obesity unless housed to thermal neutral condition [19,20]. Although diminished thermogenesis was observed in mice lacking CPEB2 (CPEB2-KO) or *Ucp1L* (*Ucp1ΔL*) at the age of 4–5 months, impaired metabolic activity, as measured by ^18^F-FDG uptake, was only found in CPEB2-KO BAT [13]. This led us to investigate whether CPEB2-KO and *Ucp1ΔL* mice may be susceptible to developing obesity as they age.

By monitoring body weight, we found increased weight gain in CPEB2-KO mice compared to their wild-type (WT) littermates, whereas no significant difference was observed in *Ucp1ΔL* mice. Using two conditional knockout (cKO) models of CPEB2 and AAV rescue experiment, we confirmed that the loss of CPEB2 specifically in BAT contributes to the increased body weight observed in CPEB2-KO mice. Therefore, we conducted RNA-sequencing to analyze the transcriptome of CPEB2-deficient BAT, revealing significant upregulation of genes involved in muscle development and function.

Lineage tracing experiments have revealed that both brown adipocytes and myoblasts originate from myogenic factor 5 (Myf5)-expressing mesodermal precursors during embryonic development [21,22], with the fate switch to brown adipocytes being contingent upon PRDM16 [[23], [24], [25]]. In CPEB2-KO BAT, there was a significant downregulation of PRDM16 expression at the protein level. To investigate whether diminished PRDM16 expression is the primary factor driving the elevated myogenic characteristic and weight gain, we introduced PRDM16 ectopically into BAT. This led to altered expression not only of muscle-related genes but also immune-related genes, along with a significant reduction in weight gain in CPEB2-KO mice. Thus, the translational activation of *Prdm16* by CPEB2 is crucial for suppressing myogenic characteristics and maintaining metabolic activity in mature brown adipocytes, thereby conferring resistance to obesity.

## 2 MATERIALS and METHODS

### 2.1 Animals and genotyping

Animal study was conducted under the approval of the Institutional Animal Care and Utilization Committee of Academia Sinica (protocol no.: 12-03-338). UCP1-KO (JAX:003124), *Nestin*-Cre (JAX:003771) and *aP2*-Cre (JAX:005069) mice [[26], [27], [28], [29]] were purchased from the Jackson Laboratory. The generation of CPEB2-KO and *Ucp1ΔL* mice on a C57BL/6 genetic background was previously described [10,13]. CPEB2-WT and -KO mice were obtained from heterozygous crosses. Conditional knockout of *Cpeb2* (CPEB2-cKO) in neuron/glia or brown/white adipocytes was achieved by breeding floxed *Cpeb2* (*Cpeb2*^f/f, +/+^) female mice with *Cpeb2*^f/f,^ ^*nestin*-cre/+^ or *Cpeb2*^f/f,^ ^*aP2*-cre/+^ male mice, respectively. Mice were housed under a 12-h light/dark cycle in a temperature-controlled room (22–24 °C) with *ad libitum* access to water and food (LabDiet 5058 with 21.6% of calories from 9% fat). For experiments in Figure 2B and Supplementary Fig. 2A, mice were fed with a high fat diet (HFD, TestDiet 58Y1 with 60% of calories from 35% fat). Genotypes were determined via PCR of tail biopsies as previously described [10,13].

### 2.2 Antibody information

The monoclonal CPEB2 antibody was generated in house [30,31]. The other antibodies are UCP1 (ab10983) from Abcam, PRDM16 (AP23499PU-N) from Origene, α-tubulin (T5168), GAPDH (MAB374) and goat anti-mouse IgG HRP-conjugated (AP124P) from Sigma–Aldrich, GFP (GFP-1020) from Aves Labs, GAPDH (GTX100118) from GeneTex, flag-tag (#2368) and goat anti-rabbit IgG HRP-conjugated (7074p2) from Cell Signaling, donkey anti-mouse IgG Alexa 488 (A21202), anti-rabbit Alexa 594 (A21207) and anti-chicken Alexa 488 (A78948) from Invitrogen.

### 2.3 Measurements of food intake, body weight and composition

Food intake was measured daily for 10 or 21 consecutive days for 3.5-mo-old or 8.5-mo-old mice, respectively. Body weight was measured every 2 or 4 weeks. Body composition of lean and fat mass was determined using the Bruker Minispec LF50 TD-NMR analyzer.

### 2.4 Measurements of body and BAT temperature

Surface thermal images surrounding interscapular BAT (iBAT) were captured using NEC F30S infrared thermography and then used to calculate the average temperature. Core body temperature was recorded using a rectal thermometer probe.

### 2.5 Homecage activity

Home-cage activity was recorded by using CCTV cameras equipped with night-vision capability and analyzed by use of Clever System HomeCageScanTM3.0.

### 2.6 Tissue collection, RNA extraction and Western blot analysis

Mice were euthanized with isoflurane inhalation prior to tissue isolation. Harvested tissues were snap-frozen in liquid nitrogen and stored at −80 °C until RNA or protein preparation. Total RNA was extracted using TRIzol reagent according to the manufacturer's protocol (Invitrogen). For immunoblotting, tissues were homogenized on ice in a lysis buffer containing 20 mM HEPES, pH 7.5, 250 mM sucrose, 10 mM KCl, 1.5 mM MgCl_2_, 1 mM EDTA, 0.5% sodium deoxycholate, 0.1% SDS, 1% Triton X-100, and 1X protease inhibitor cocktail mix (Roche). The homogenate was then centrifuged at 15,000×*g* for 15 min at 4 °C. The protein concentration of collected supernatants was determined using the Pierce BCA Protein Assay Kit. Samples with equal amount of protein were denatured at 95 °C for 5 min, separated on a 10% SDS-PAGE, and then transferred to polyvinylidene fluoride (PVDF) membrane (Millpore). The membrane was incubated with the primary antibodies followed by horseradish peroxidase (HRP)-conjugated secondary antibodies and detection with chemiluminescence substrates (P90720, Millpore).

### 2.7 Quantitative RT-PCR (RT-qPCR)

RNA samples were reverse-transcribed into cDNA using random primers and ImProm-II reverse transcriptase (Promega). The resulting cDNA samples were subjected to qPCR analysis using the Universal Probe Library in the LightCycler 480 system (Roche). Relative expression levels of designated targets were calculated using the comparative threshold cycle method, with *Gapdh* mRNA as the reference. Primer sequences are listed in Supplementary Table 1.

### 2.8 Histology and immunofluorescence staining

For histological analysis and immunostaining, mice were euthanized with isoflurane inhalation before perfusion with 50 ml phosphate buffered saline (PBS), followed by 50 ml 4% formaldehyde. Collected tissues were post-fixed in 4% formaldehyde overnight and embedded in paraffin. Tissue sections after the removal of paraffin were then stained with hematoxylin and eosin [32] by the institutional Pathology Core staff. Lipid droplet size and the number of nuclei in visceral white adipose tissue (WAT) were quantified by using MetaMorph software. For immunohistochemistry staining, BAT slices were incubated with xylene for 15 min twice to remove paraffin, immersed in 100%, 75% and 50% ethanol for 5 min twice at each concentration, and then rinsed with water for 10 times. For immunostaining of CPEB2 and UCP1, rehydrated slices were immersed in 0.1% Sudan Black B in 70% ethanol for 20 min to reduce autofluorescence [33], followed by three washes of PBST (0.02% Tween-20 in PBS). For immunostaining of GFP and flag tag (i.e., flag-PRDM16), rehydrated sections were heated in a pressure cooker for 5 min in the antigen retrieval buffer (10 mM Tris and 1 mM EDTA, pH 9.0), washed with running water for 5 min, and then bathed in water for another 5 min, followed by two washes of PBS. Unless specified, the following procedures were conducted at room temperature with three washes of PBS between changes of reagents. BAT sections were permeabilized with 0.1% Triton X-100 in PBS for 10 min, blocked in 5% bovine serum albumin (BSA) for 1 h and then incubated with primary antibodies in 1% BSA at 4 °C overnight. On the next day, after 2-h incubation of fluorophore-conjugated secondary antibodies and Hoechst 33342 in 1% BSA, slices were washed with PBS three times before mounting with ProLong Gold Antifade reagent (Thermo Fisher Scientific). Images were acquired by LSM 780 confocal microscope (Zeiss).

### 2.9 Cell culture and luciferase reporter assay

Various lengths of the *Prdm16* 3′-UTR were PCR-amplified from mouse BAT cDNA using a combination of one of the sense primers, 5′-CGGACTAGTTGCTGTCCCTCTCTGAAGAC-3′ or 5′-CGGACTAGTCAGGCTACGCTGGGATCCGTGA-3′, and one of the antisense primers, 5′-ACGCGTCGACGAAAAACACACAGATTTATTGCATA-3′ or 5′-ACGCGTCGAC TTTATTCACGGATCCCAGCGTAGCCT-3'. The resulting DNA fragments were digested with SpeI and SalI and then cloned into the pGL3-promoter vector linearized with XbaI and SalI (Promega). HEK293T cells were cultured in DMEM with 10% fetal bovine serum. The cells were transfected with plasmids expressing firefly luciferase appended with various *Prdm16* 3′-UTR sequences and *Renilla* luciferase, along with enhanced GFP, full-length or the RNA-binding domain of CPEB2 using lipofectamine 2000 transfection reagent. Transfection was carried out overnight before harvesting the cells for the dual luciferase reporter assay (Promega).

### 2.10 Recombinant AAV production and AAV administration into iBAT

Recombinant AAV constructs expressing myc-tagged full length or the RNA-binding domain of CPEB2 were previously reported [11,13]. The mouse PRDM16 coding region was PCR-amplified using the primers: 5′-GCGAATTCGGCCATGGCCCCCAGCTTGGAT-3′ and 5′-CCGCTCGAGTTAGAGGTGGTTGATGGGGTTA-3′, and then cloned into EcoRI- and XhoI-linearized cDNA3.1-3Xflag plasmid. The resulting 3Xflag-PRDM16 DNA fragment was excised by NheI and PmeI, and then cloned into XbaI- and HincII-digested pAAV-MCS vector. Recombinant AAV viruses were produced by the institutional AAV core, along with the pHelper and AAV2/8 plasmids, and their titers were determined by qPCR as described [34]. Mice were anesthetized with 1.2% Avertin and shaved ∼2 cm below the head. Subsequently, approximately 2 × 10^10^ vg (vector genome) of recombinant AAV in 30 μl PBS was bilaterally injected into the BAT. Mice receiving AAV delivery were monitored for their body weight gain.

### 2.11 RNA sequencing

Isolated iBAT RNA samples were submitted to Welgene Inc. (Taiwan) and their quantities and qualities were determined by using the ND-1000 spectrophotometer (Nanodrop Technology) and Bioanalyzer 2100 with the RNA 6000 Nano kit (Agilent Technology). The SureSelect XT HS2 mRNA Library Preparation kit (Agilent Technology) was used to construct cDNA library, which was then size-selected by using AMPure XP beads (Beckman Coulter). The sequence was determined using Illumina's sequencing-by-synthesis technology. Sequencing data (FASTQ reads) were generated using Welgene Biotech's pipeline based on Illumina's base-calling program BCL Convert v4.2.4. The RNA-seq data have been deposited in the GEO database under accession number GSE242882.

### 2.12 Polysome profiling and RNA-immunoprecipitation (RNA-IP)

The protocols used were described previously [13]. Briefly, BAT was lysed in polysome buffer containing 25 mM Hepes (pH 7.5), 25 mM NaCl, 5 mM MgCl_2_, 0.3% NP-40, 100 μg/ml cycloheximide, 0.5 mM DTT, 20 U/ml RNase inhibitor and 1X protease inhibitor cocktail) and then centrifuged at 15,000×*g* for 15 min at 4 °C. The resulting supernatant was layered onto a 10 ml linear 15–50% (w/v) sucrose gradient of 10 ml and centrifuged in a SW41 rotor at 37,000 rpm for 2 h. Polysomal fractions were collected from the top of the sucrose gradient in 800 μl per fraction. For RNA-IP, BAT was lysed in IP buffer containing 50 mM Hepes (pH 7.4), 150 mM NaCl, 1 mM MgCl₂, 0.5% Triton X-100, 10% glycerol, 1 mM DTT, 1X protease inhibitor cocktail, and RNase inhibitor. Lysates were centrifuged at 15,000×*g* for 15 min, and the supernatant was divided and incubated with protein G beads bound to either CPEB2 or control IgG for 3 h. The precipitated samples were either immunoblotted with CPEB2 or eluted using buffer containing 100 mM Tris (pH 8), 10 mM EDTA, and 1% SDS. Both the eluted samples and the sucrose gradient fractions were treated with 100 μg/ml Proteinase K for 30 min at 37 °C, followed by phenol/chloroform extraction and ethanol precipitation to obtain RNA for RT-qPCR analysis.

### 2.13 Analysis of GWAS data from Taiwan, UK and Japan Biobanks

The websites of the Taiwan Biobank (https://taiwanview.twbiobank.org.tw/), UK Biobank (https://www.ebi.ac.uk/gwas/) and Japan Biobank (https://pheweb.jp/) were used to search for potential associations of *CPEB2*, *PRDM16* and *UCP1* with human body weight and obesity (BMI).

### 2.14 Data presentation and statistical analysis

All data are expressed as mean ± SEM. Statistical analyses involved use of GraphPad Prism. Sample sizes and statistical methods (Student's *t* test or ANOVA) for experiments are in figure legends.

## 3 Results

### 3.1 CPEB2-KO female mice show an obese phenotype as they age

**Figure 1:**
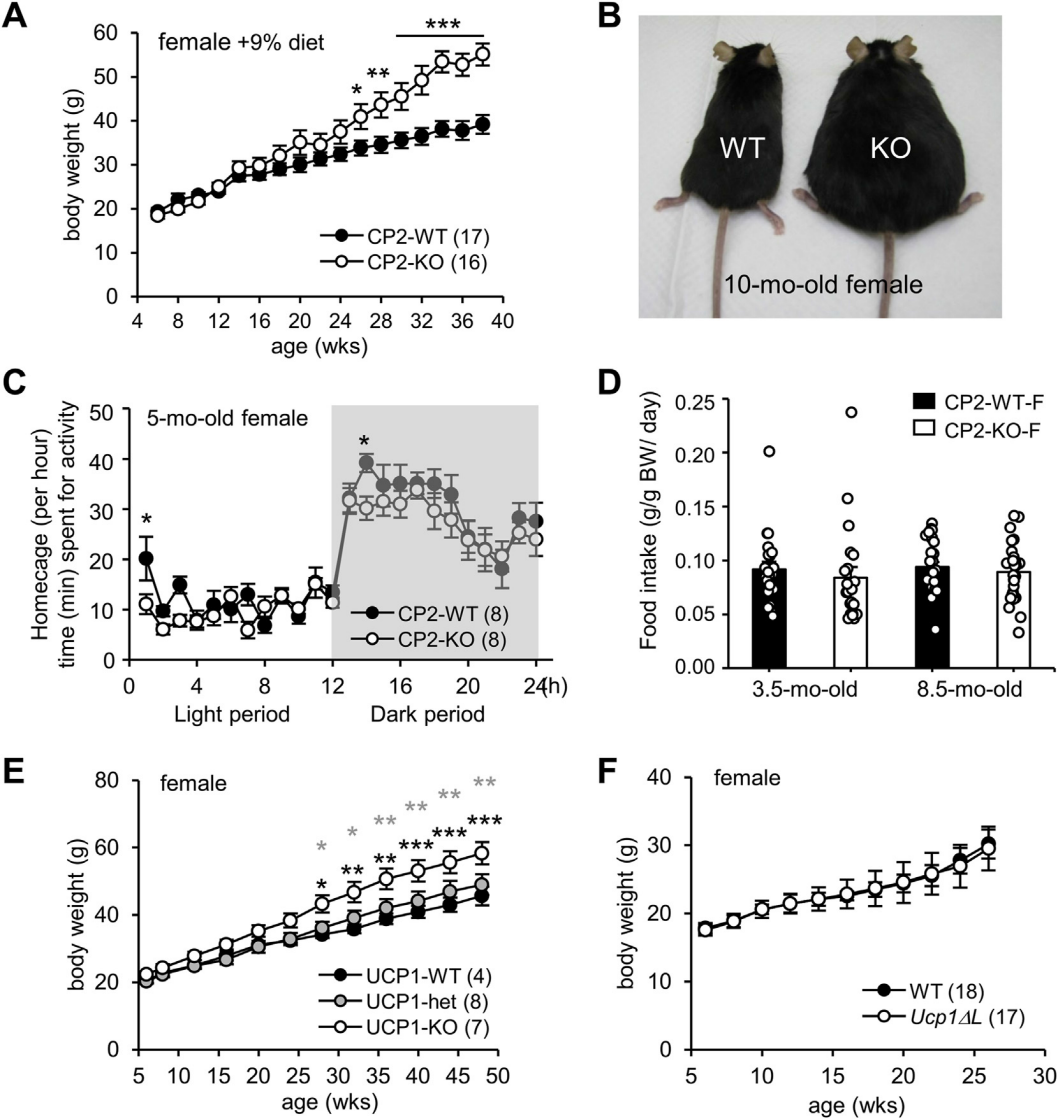
**Obesogenic phenotype of CPEB2-knockout (KO) female mice is caused by hypometabolism. (A)** Body weight of CPEB2-WT and -KO female littermates fed a 9% fat diet. **(B)** Photograph of WT and KO female littermate at 10 months (mo) old. **(C)** Homecage activity of 5-mo-old females monitored for 48 h under 12-h light/dark cycles. The time spent (min) per hour in spontaneous activities, including drinking, eating, grooming, rearing, hanging and walking, is indicated. **(D)** The average daily food consumed (in g) per gram body weight by 3.5-mo-old or 8.5-mo-old female mice were recorded for consecutive 10 days or 21 days, respectively (3.5-mo-old, n = 21 for both groups of mice; 8.5-mo-old, 28 WT and 26 KO mice). **(E)** Body weight of UCP1-WT, -heterozygous and -KO female littermates fed a 9% fat diet. **(F)** Body weight of WT and *Ucp1ΔL* female littermates fed a 9% fat diet. Data are mean ± SEM. ∗*P* < 0.05, ∗∗*P* < 0.01, ∗∗∗*P* < 0.001, two-way ANOVA with Fisher's LSD *post-hoc* test. Numbers in parentheses denote the number of mice in each group.

We previously reported that approximately 70% CPEB2-KO mice in a C57BL/6 genetic background died early postnatally due to respiratory defects [9,10]. Notably, the weight of surviving female but not male KO mice receiving the LabDiet 5058 (9% fat, 21.6% of calories from fat) began to be significantly greater than their WT littermates at 7 months (mo) of age and remained high as they aged (Figure 1A). The KO females could weigh 50% more than WT females, on average, at 9 mo old (Figure 1A); at the extreme, KO females could weigh twice as much as WT littermates (Figure 1B).

**Figure 2:**
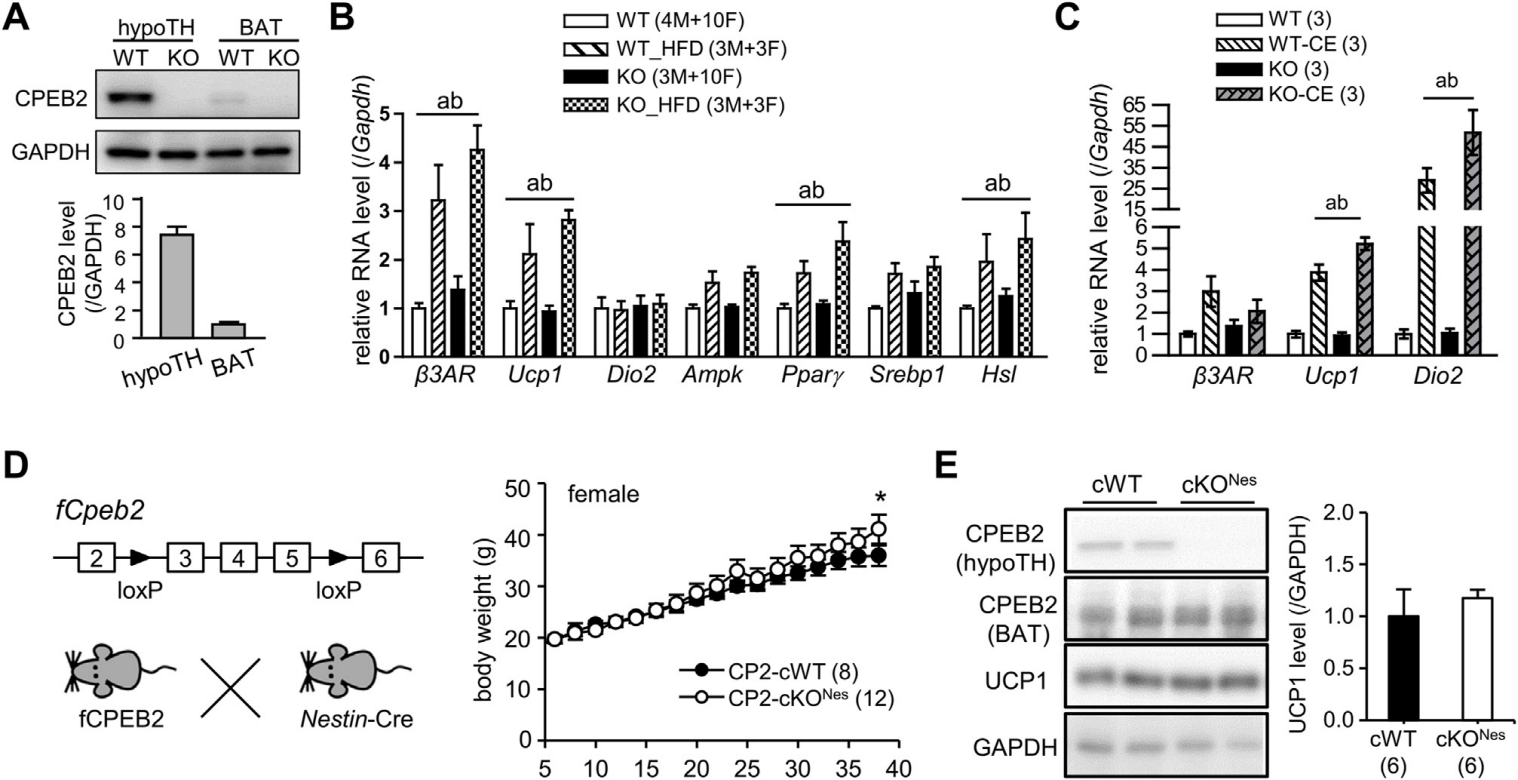
**Adaptive thermogenesis-evoked transcriptional upregulation is normal in CPEB2-KO mice. (A)** Western blot analysis of CPEB2 level in hypothalamus (hypoTH) and BAT (n = 3 male mice). **(B)** RT-qPCR analysis of expression of genes associated with thermogenesis and lipid metabolism in BAT of WT and KO mice fed 9% fat diet or a high-fat diet (HFD; n = male (M)/female (F), WT: 4/10; WT_HFD: 3/3; KO: 3/10; KO_HFD: 3/3). Annotations indicate significant effects of a = diet and b = diet × genotype interaction, two-way ANOVA. **(C)** RT-qPCR analysis of expression of genes associated with thermogenesis in BAT of WT and KO male mice after 5-h cold exposure (CE, n = 3 per group). Annotations denote significant effects of a = temperature and b = temperature × genotype interaction, by two-way ANOVA. β3AR, β3 adrenergic receptor; Ucp1, uncoupling protein 1; Dio2, iodothyronine deiodinases II; Ampk, AMP-activated protein kinase; Pparγ, peroxisome proliferator-activated receptor γ*;* Srebp1, sterol regulatory element-binding transcription factor 1; Hsl, hormone-sensitive lipase. Data are mean ± SEM. **(D)** Body weight of cWT (*Cpeb2*^f/f^) and neuron/glia-specific cKO (*Cpeb2*^f/f, Nes-cre/+^) female mice (cWT/cKO, n = 8/12). **(E)** BAT UCP1 levels in cWT and cKO^Nes^ mice at 10 mo old (n = 6 per group).

Ovarian estrogen has anti-obesity effects in mammals, with deficiency exacerbating the obesogenic phenotype [35,36]. Since the age-related obesity in CPEB2-KO mice was female-prominent, we analyzed ovarian morphology (Supplementary Figure 1A), estrous cycle (Supplementary Figure 1B) and serum levels of estradiol-17β (Supplementary Figure 1C) and progesterone (Supplementary Figure 1D) and found no obvious defects in KO female mice. In addition, we found that KO female mice could give a birth, but they failed to nurse pups likely due to maldeveloped mammary gland [12]. Thus, WT and KO mice were obtained from heterozygous mating and analyzed for their homecage activities and food intake before showing difference in body weight.

Female mice lacking CPEB2 showed slightly reduced motility in the homecage (Figure 1C, 5 mo old), yet their food intake remained normal (Figure 1D, 3.5 mo old). They became hyperphagia only after the development of obesity (Figure 1D, 8.5 mo old). Upon normalization by body weight, the amount of food consumed by both WT and KO mice appeared similar regardless of age (Figure 1D), which suggests that the increased food intake in overweight KO females likely meets the energy demand associated with their rising body mass, rather than being the primary cause of obesity. Previously, we found that energy expenditure in both male and female CPEB2-KO and *Ucp1ΔL* mice was attenuated when housed at 30 °C (i.e., the thermoneutral temperature for mice) and challenged with a β3AR agonist, CL316243. This thermogenic defect arises from the loss of CPEB2-activated *Ucp1L* translation, leading to the production of ∼50% less UCP1 protein [13]. Because a previous study reported that UCP1-KO female mice but not males also exhibited obesity after 6 months of age under a high fat diet (HFD, 41.9% of calories from fat) [37], we monitored their body weight under LabDiet 5058 (9% fat, 21.6% of calories from fat) and found increased body weight in UCP1-KO but not in UCP1-heterozygous (Figure 1E) and *Ucp1ΔL* female mice (Figure 1F). Hence, the ∼50% reduction in the UCP level in *Ucp1ΔL* mice and UCP1-heterozygous mice is insufficient to cause elevated weight gain. Therefore, the obesogenic phenotype in CPEB2-KO female mice is unlikely caused by estrogen deficiency or reduced UCP1 expression.

### 3.2 Normal adaptive thermogenesis-evoked transcription in CPEB2-KO mice

Adaptive thermogenesis in response to nutrient or cold temperature is under the control of hypothalamus-governed sympathetic circuits via the release of norepinephrine binding to β3ARs in brown adipocytes, which initiates a cascade of regulatory processes to augment UCP1 synthesis and heat production [14,38,39]. Activation of β3ARs leads to cyclic AMP-stimulated protein kinase A (PKA), which then induces hormone-sensitive lipase (HSL)-triggered lipolysis to acutely increase UCP1 function with elevated level of free fatty acids. Chronically, PKA upregulates *Ucp1* gene transcription via multiple transcription factors, including peroxisome proliferator-activated receptor (PPAR)γ and PPARγ co-activator (PGC)1α. Because CPEB2 is abundantly expressed in the brain [10], we compared its expression in the hypothalamus and BAT and found the level of CPEB2 greater in the hypothalamus than BAT, by ∼7.5-fold (Figure 2A). To determine whether the signaling from hypothalamus-sympathetic nerves to BAT is defective, we analysed transcript profiles in BAT isolated from CPEB2-WT and -KO mice on a regular diet (9% fat) or a HFD (35% fat). We found a comparable increase in mRNA levels of *β3AR*, *Ucp1*, *Pparγ*, and *Hsl* in HFD-challenged WT and KO BAT (Figure 2B). Similarly, cold-induced synthesis of *Ucp1* and iodothyronine deiodinases II (*Dio2*) RNAs was similar in KO and WT mice (Figure 2C). Thus, the adaptive thermogenic β3AR signaling from hypothalamus-sympathetic nerve to BAT appears normal in CPEB2-KO mice. In line with the role of CPEB2 on promoting *Ucp1L* translation in response to β3AR signaling [13], the UCP1 protein level was also reduced in CPEB2-deficient BAT after HFD feeding (Supplementary Figure 2A) or cold exposure (Supplementary Figure 2B).

### 3.3 CPEB2 deficiency in adipose tissues induces female mouse obesity

**Figure 3:**
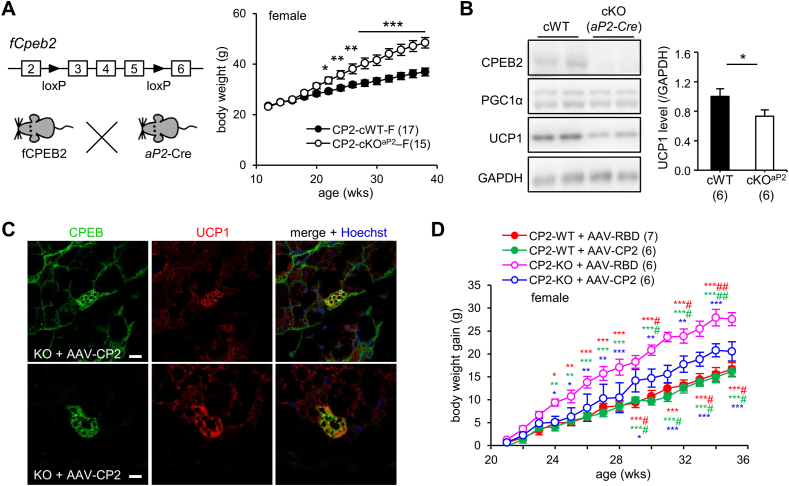
**Ectopic expression of CPEB2 in the KO BAT increases UCP1 synthesis and reduces weight gain. (A)** Body weight of cWT and adipose-specific cKO (*Cpeb2*^f/f, aP2-cre/+^) female mice. Data are mean ± SEM. ∗*P* < 0.05, ∗∗*P* < 0.01, ∗∗∗*P* < 0.001, two-way ANOVA. **(B)** Western blot analysis of BAT UCP1 protein level in cWT and cKO^aP2^ mice at 10 mo old. ∗*P* < 0.05, Student's *t* test. **(C)** CPEB2 WT and KO mice were transduced with adeno-associated virus (AAV) expressing full-length (AAV-CP2) or RNA-binding domain (AAV-RBD) of CPEB2. Immunostaining of CPEB2 and UCP1 in KO BAT transduced with AAV-CP2 for 2 weeks, with Hoechst staining of nuclei. Scale, 10 μm. **(D)** Body weight gain of WT and KO female mice (20-22-week-old) after transduction with AAV-CP2 or AAV-RBD. Data are mean ± SEM. ∗*P* < 0.05, two-way ANOVA. with Fisher's LSD *post-hoc* test. KO + RBD compared with KO + CP2 (blue∗); with WT + RBD (red∗); and with WT + CP2 (green∗). KO + CP2 compared with WT + RBD (red#); with WT + CP2 (green #). ∗ #*P* < 0.05, ∗∗ ##*P* < 0.01, ∗∗∗*P* < 0.001, two-way ANOVA with Fisher's LSD *post-hoc* test. Numbers in parentheses denote the number of mice in each group. (For interpretation of the references to color in this figure legend, the reader is referred to the Web version of this article).

To further elucidate whether CPEB2 deficiency in BAT is the key factor leading to UCP1 downregulation and increased body weight, we generated conditional knockout (cKO) mice by crossing fCPEB2 mice with *Nestin-*Cre or *aP2*-Cre transgenic mice to achieve conditional knockout of *Cpeb2* in pan neurons/glia (cKO^Nes^) or adipocytes (cKO^aP2^), respectively. Unlike global KO mice, neither cKO^Nes^ nor cKO^aP2^ females exhibited any mortality. In line with our hypothesis, CPEB2-cKO^Nes^ female mice maintained normal body weight until 10-mo-old (Figure 2D) and UCP1 protein level (Figure 2E), whereas CPEB2-cKO^aP2^ female mice weighed significantly more than their conditional WT (cWT) littermates beginning at 5.5-mo old (Figure 3A) and showed reduced UCP1 expression (Figure 3B). Thus, the loss of CPEB2 function in adipose tissues emerges as the primary driver of the obesogenic phenotype in the global KO mice.

### 3.4 Ectopic expression of CPEB2 in BAT improves UCP1 expression and mitigates obesity in KO female mice

We previously reported that CPEB2-KO mice with reduced UCP1 expression have enlarged brown adipocytes as they age [13]. Despite the increased mass of visceral WAT in obese KO female mice at ∼8–9 mo old (Supplementary Figure 3A), the size of lipid droplets and the morphology of white adipocytes remained comparable to those in WT mice (Supplementary Figure 3B). Since *aP2-Cre* mice express Cre recombinase in both brown and white adipocytes [28], we first investigated whether the loss of CPEB2 in BAT is the primary cause of elevated body weight. AAV expressing full-length (AAV-CP2) or the RNA-binding domain (AAV-RBD) of CPEB2 was injected into the interscapular BAT (iBAT) of both WT and KO mice. The RBD of CPEB2 binds to RNA but is unable to regulate translation, serving as a control to ensure that AAV infection did not affect UCP1 expression [13]. Two weeks after AAV-CP2 transduction, an increase in UCP1 signal was observed in KO brown adipocytes expressing exogenous CP2 (Figure 3C). The weight gain of female WT and KO mice (5-mo-old) after receiving ectopic CP2 or RBD in iBAT was monitored for 15 weeks, with an additional injection of AAV-CP2 or AAV-RBD 8 weeks later. Despite CPEB2-KO mice still showing greater weight gain after AAV transduction, those with AAV-CP2 but not AAV-RBD, exhibited significantly reduced weight gain. Moreover, there were no weight differences among the three groups of mice, KO mice with AAV-CP2 and WT mice with AAV-CP2 or AAV-RBD (Figure 3D).

### 3.5 CPEB2-KO male mice also show increased body weight gain

**Figure 4:**
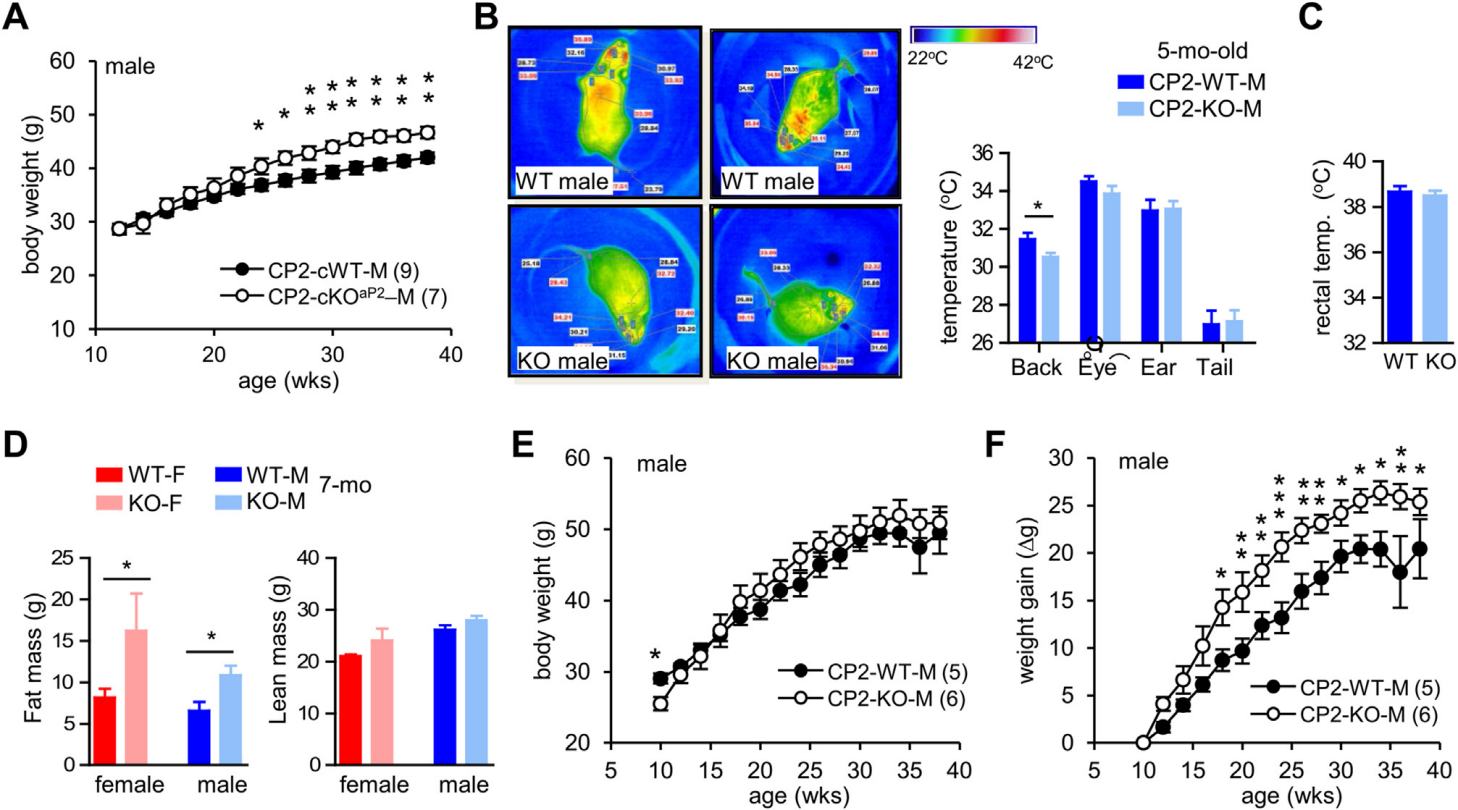
**CPEB2-KO mice show impaired BAT thermogenesis and increased body fat composition. (A)** Body weight of CPEB2-cWT and -cKO^aP2^ male littermates fed a 9% fat diet. **(B)** Infrared thermography images of CPEB2 WT and KO male mice at 5 mo old (n = 6–8). The thermal images were used to calculate surface back temperature. **(C)** The core body temperature of mice used in (B) determined by a rectal thermometer. **(D)** Fat and lean mass in WT and KO mice of both sexes determined in mice at 7 mo old by MRI (female/male, n = 5–6/6-7). **(E)** Body weight and **(F)** weight gain of CPEB2-WT and -KO male littermates fed a 9% fat diet. Data are mean ± SEM. ∗*P* < 0.05, ∗∗*P* < 0.01, two-way ANOVA for (A, E, F) and Student's *t* test for (B–D).

The primary reason of obesity in KO female mice is the absence of CPEB2 in iBAT (Figure 3D). Similar to females, we also found that CPEB2-KO males have reduced *Ucp1L* translation, blunted thermogenesis and decreased ^18^F-FDG uptake in iBAT [13]. However, they did not manifest overt overweight. Due to their early respiratory stress [9,10], surviving KO mice of both sexes usually display smaller body sizes than WT littermates after weaning. We speculated that may be the reason confound with body size change in KO male mice, so we measured the body weight in CPEB2-cKO^aP2^ male mice, revealing a significant increase starting at 6-mo old (Figure 4A). To further corroborate BAT dysfunction in CPEB2-KO male mice, infrared thermography was used to detect reduced surface temperature in the back of CPEB2-KO male mice, where the iBAT is located (Figure 4B), yet their core body temperature appeared normal (Figure 4C). Moreover, analysis using magnetic resonance imaging (MRI) to assess body composition indicated a higher percentage of body fat mass in KO mice of both sexes (Figure 4D). Because the overall body weight was notably lower in KO male mice at the young age (Figure 4E), calculations of weight gain revealed a significant increase in CPEB2-KO male mice (Figure 4F). These findings suggest that the molecular defects identified in our previous and current studies are associated with changes in body weight in male mice. However, the reasons for the more pronounced effects in females remain unclear.

### 3.6 CPEB2 binds to and activates translation of *Prdm16* mRNA

**Figure 5:**
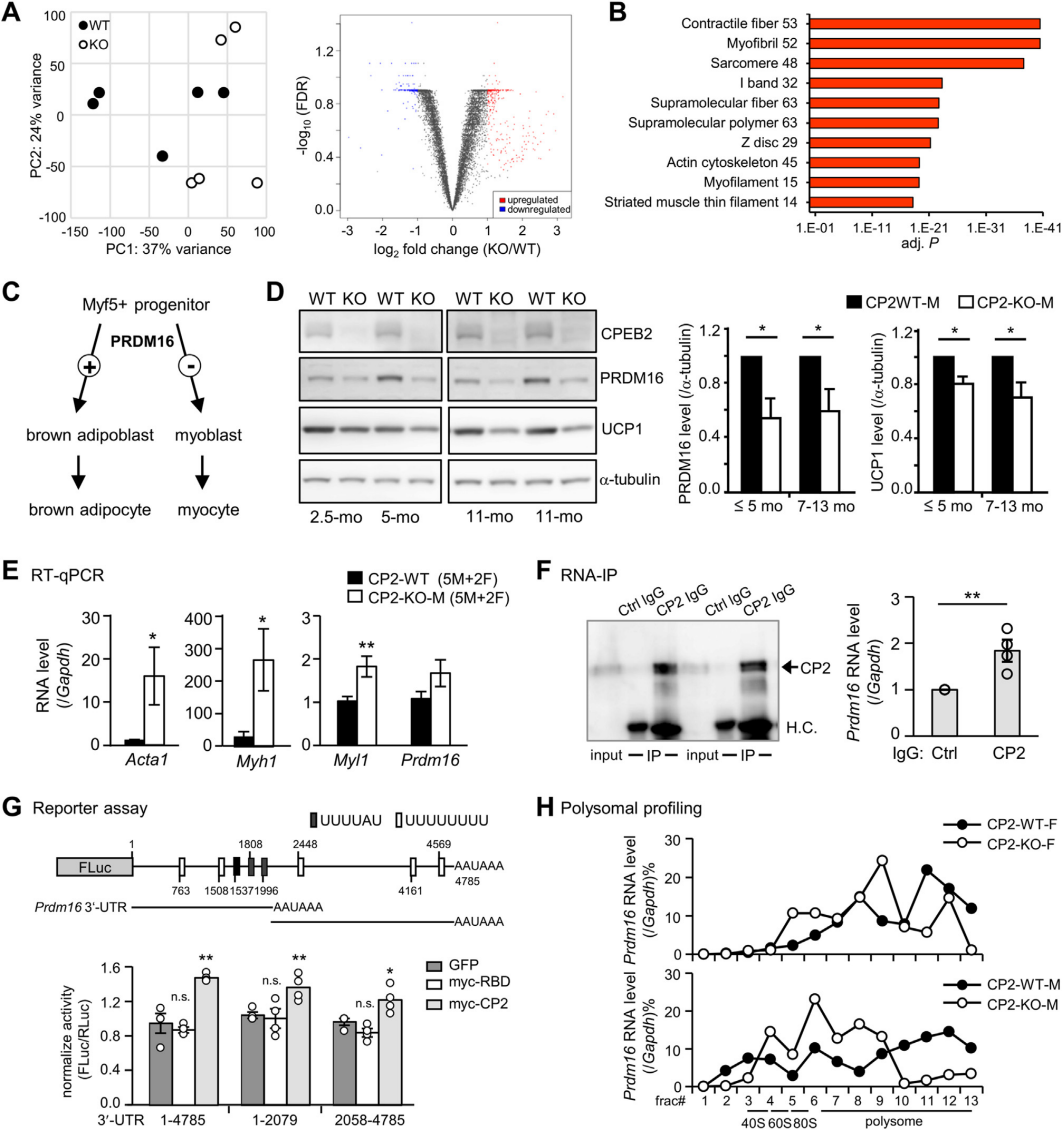
**CPEB2 deficiency impairs *Prdm16* translation and fails to suppress muscle gene expression in BAT. (A)** BATs isolated from CPEB2-WT and -KO mice (n = 5) were used for RNA-seq. The principal component analysis (PCA) plot in the left shows different distribution between CPEB2-WT and -KO BATs. The volcano plot shows the red dots and blue dots respectively representing upregulated and downregulated transcripts in CPEB2-KO BATs. **(B)** The top 10 gene ontology (GO) categories are listed in the right ranking with their *P* values. The numbers of differentially expressed transcripts in each category are also listed. **(C)** PRDM16 switches the cell fates of Myf5^+^ precursors differentiating to brown adipoblasts or myoblasts. **(D)** CPEB2-WT and -KO BATs from young (≤5 mo, n = 5 per group) and old (7–13 mo, n = 4 per group) male mice were used for western blotting and quantified relative to α-tubulin level. **(E)** RT-qPCR analysis of *Acta1*, *Myh1*, *Myl1* and *Prdm16* mRNA levels (relative to *Gapdh*) in BATs collected from mice of 2-mo-old (1M+1F), 5-mo-old (2M) and 11-mo-old (2M+1F). **(F)** RNA immunoprecipitation (RNA-IP) was conducted using BAT lysates from adult male mice with control (Ctrl) or CPEB2 (CP2) IgG. The experiment was repeated four times and the precipitated samples were analyzed by RT-qPCR to quantify the RNA levels of *Prdm16* and *Gapdh* by RT-qPCR. H.C., heavy chain. **(G)** Dual luciferase reporter assays were performed across 4 independent experiments. The 4785-bp mouse *Prdm16* 3′-UTR contains 3 conventional CPEs (UUUUAU, black boxes) and 5 U-rich sequences (white boxes). Reporter plasmids, firefly luciferase (FLuc) appended with different lengths of *Prdm16* 3′-UTR and *Renilla* luciferase (RLuc), were co-transfected with the plasmid expressing GFP, or myc-tagged full-length (CP2) or RNA-binding domain (RBD) of CPEB2 into 293T cells. **(H)** BATs from WT and KO mice of both sexes were fractionated on a sucrose gradient. RT-qPCR analysis of the polysomal distribution of *Prdm16* mRNA with RNA isolated from each fraction. Data are mean ± SEM. ∗*P* < 0.05, ∗∗*P* < 0.01, ∗∗∗*P* < 0.001, n.s., not significant, Student's *t* test. (For interpretation of the references to color in this figure legend, the reader is referred to the Web version of this article).

Despite attenuated β3AR-activated thermogenesis in both CPEB2-KO and *Ucp1ΔL* mice, we found that the uptake of ^18^F-FDG by iBAT is impaired only under CPEB2 deficiency, at both room temperature and after cold exposure [13]. Therefore, the decreased metabolic activity in CPEB2-KO BAT cannot be solely attributed to diminished UCP1 expression, which likely accounts for elevated weight gain only in CPEB2-KO mice (Figure 1A, F). To reveal why CPEB2-deprived BAT is less metabolically active, CPEB2-WT and -KO mice (4–5 mo old) were food-deprived for 5–6 h to minimize food-induced adaptive thermogenesis before collecting their BAT for RNA isolation and sequencing (RNA-seq). Transcriptomic data were analyzed using the on-line tool, integrated differential expression and pathway analysis (iDEP) and Database for Annotation, Visualization and Integrated Discovery (DAVID) [[40], [41], [42]]. Principal component analysis (PCA) revealed transcriptomic variabilities between and within the WT and KO groups (Figure 5A). All identified genes were displayed in the volcano plot, where differentially expressed genes (DEGs, defined by ≥ ± 2-fold change and a false discovery rate (FDR) ≤ 0.5) were labeled in red for 290 upregulated genes and in blue for 240 downregulated genes in CPEB2-KO BATs (Figure 5A and Supplementary Table 2). Gene ontology (GO) and Kyoto Encyclopedia of Genes and Genomes (KEGG) pathway analyses of the upregulated (Figure 5B) and downregulated DEGs (Supplementary Figure 4) were conducted using DAVID [41,42]. DEGs in the GO categories and KEGG pathways are listed (Supplementary Table 3). Remarkably, the 290 upregulated genes in CPEB2-KO BAT are mostly associated with muscles (Figure 5B). Brown adipocytes and myocytes are originated from Myf5^+^ progenitors, and PRDM16 regulates the bidirectional switch between myoblasts and brown adipoblasts (Figure 5C) [23,43]. PRDM16 promotes brown adipocyte gene expression and differentiation, while its depletion results in muscle differentiation in culture [23]. Therefore, we speculated that CPEB2 deficiency leads to a decrease in *Prdm16* translation, resulting in increased muscle-related GO categories and transcripts in CPEB2-KO BAT transcriptomes (Figure 5B). Western blotting was applied to examine the expression of PRDM16 in CPEB2-WT and -KO BATs at various ages. The quantified results showed reduced PRDM16 levels in BATs of young (≤5 months) and old (7–13 months) CPEB2-KO mice (Figure 5D). The decreased expression of UCP1 in CPEB2-KO BATs conforms CPEB2-mediated translational activation of *Ucp1L* in our previous research [13]. Moreover, using RT-qPCR, we confirmed the upregulation of *Acta1*, *Myh1* and *Myl1* but not *Prdm16* mRNA in KO BATs (Figure 5E). These results suggest that the loss of CPEB2 affects PRDM16 expression at the protein but not mRNA level.

To further establish the role of CPEB2 in the translation of *Prdm16* mRNA, RNA-immunoprecipitation (RNA-IP) was performed using BAT. The amount of *Prdm16* mRNA precipitated was significantly higher with CPEB2 IgG compared to the control IgG, indicating a direct interaction between CPEB2 and *Prdm16* mRNA (Figure 5F). We performed the reporter assay in 293T cells using firefly luciferase (FLuc) appended with varying lengths of mouse *Prdm16* 3′-UTR and discovered that the translation efficiency of the FLuc reporters was promoted by the full-length but not the RBD of CPEB2 (Figure 5G). Moreover, CPEB2 deficiency led to a shift of *Prdm16* mRNA toward less dense sucrose fractions (i.e., a decrease in number of ribosomes associated with *Prdm16*) in both female and male BAT (Figure 5H). Together, CPEB2 in BAT is necessary for suppressing myogenic remodeling via activating *Prdm16* translation.

### 3.7 Ectopic expression of PRDM16 in BAT alleviates weight gain in CPEB2-KO mice

**Figure 6:**
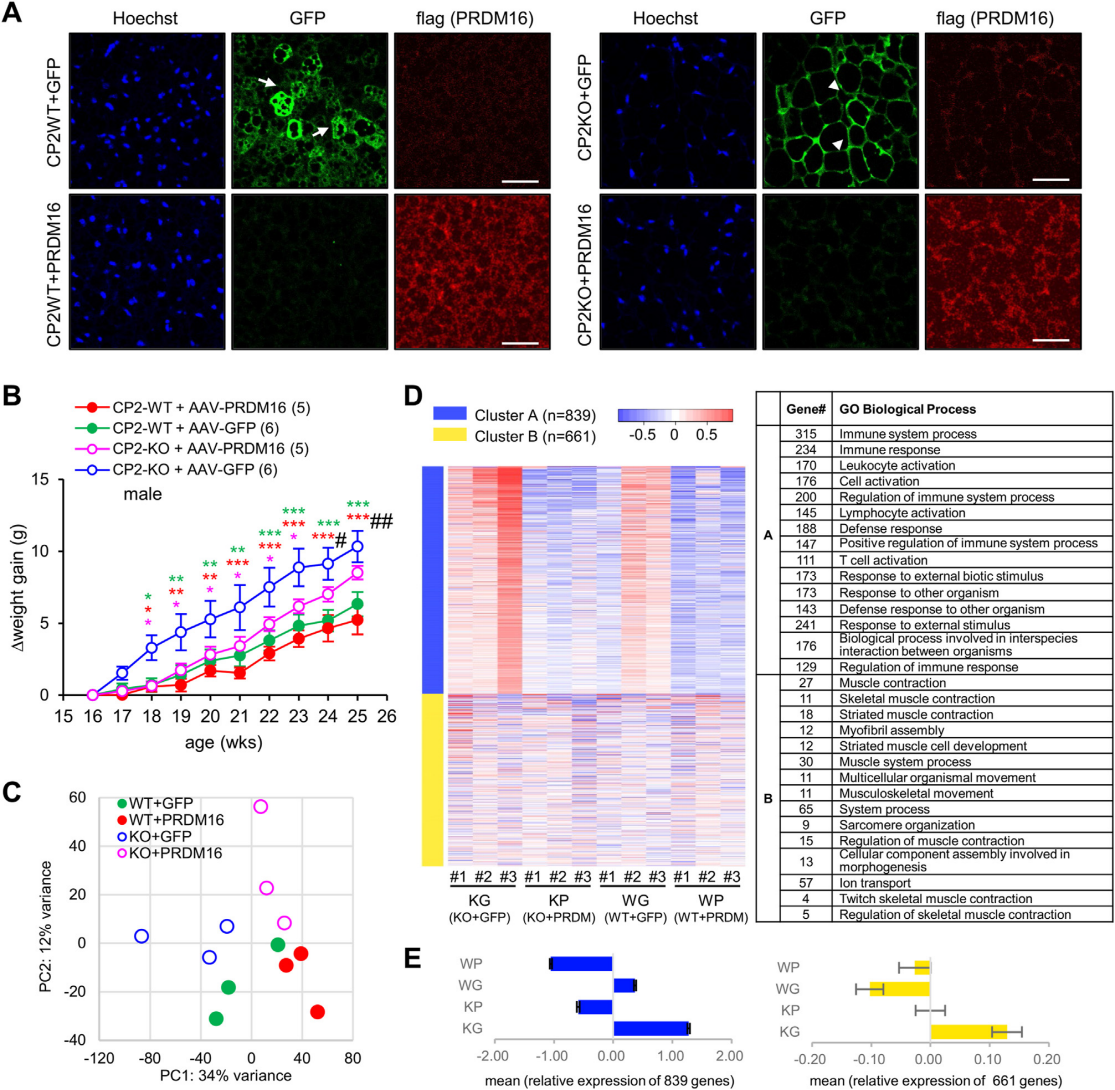
**Ectopic expression of PRDM16 in CPEB2-KO BATs reduces weight gain and muscle gene expression. (A)** The immunostaining images of CPEB2-WT and -KO BAT infected with AAV-GFP or AAV-flag-PRDM16. Scale, 50 μm. **(B)** The accumulated body weight gains after AAV infection in CPEB2-WT and -KO BATs. Numbers in parentheses denote the number of mice in each group. Color asterisks, KO + GFP compared with KO + PRDM16 (magenta); with WT + GFP (green); with WT + PRDM16 (red), and #, WT + PRDM16 vs KO + PRDM16. ∗ #*P* < 0.05, ∗∗ ##*P* < 0.01, ∗∗∗ <0.001, two-way ANOVA with Fisher's LSD *post-hoc* test. **(C)** CPEB2-WT and -KO BATs injected with AAV-GFP or AAV-flag-PRDM16 (3 mice for each group) were isolated for RNA-Seq. The PCA plot shows specified distribution between each group. **(D)** The top 1500 genes with the most variable expression were processed for *k*-means clustering analysis and grouped into 2 GO categories. The heat map is shown on the left, where shades of red and blue represent the relative level of upregulation and downregulation, respectively, among the 12 samples. The detailed pathways in the 2 categories are listed in the right panel, with the number of genes indicated for each pathway. **(E)** The standard deviations (SDs), reflecting the relative expression level of each gene after k-means clustering, were used to calculate the mean ± SEM for cluster A (839 genes) and cluster B (661 genes). (For interpretation of the references to color in this figure legend, the reader is referred to the Web version of this article).

Given the significant reduction of PRDM16 in CPEB2-KO BATs (Figure 5D), we evaluated whether ectopic expression of PRDM16 in CPEB2-deficient iBAT could alleviate weight gain in CPEB2-KO mice. Male WT and KO mice at the age of 16 weeks were injected with AAVs expressing flag-PRDM16 or GFP (Figure 6A). Their body weight was monitored weekly for 9 weeks, after which BATs were collected for immunofluorescence staining or RNA isolation. Representative images showed the expression of GFP or flag-PRDM16 in CPEB2-WT and -KO BATs. Notably, in BAT transduced with AAV-GFP, WT brown adipocytes exhibited a multilobular morphology (Figure 6A, arrows), while some KO brown adipocytes displayed a white adipocyte-like monolobular morphology (Figure 6A, arrow heads). The accumulated weight gains after AAV delivery were significantly different between the groups, showing that ectopic expression of PRDM16 in BAT reduced weight gain in CPEB2-KO mice (Figure 6B). To delineate transcriptomic changes in WT and KO BATs transduced with AAVs, BAT RNA samples were processed for RNA-seq to obtain transcript expression levels (Supplementary Table S4). The PCA plot showed distinct clustering of transcriptomes among the 4 groups of samples (Figure 6C). The heat map from *k*-means clustering analysis of the top 1500 DEGs highlighted the most affected GO biological processes related to immune function (cluster A) and muscle function (cluster B), showing that the “immune system process” with the smallest P-value ranked on the top (Figure 6D and Supplementary Table S5). Ectopic expression of flag-PRDM16 strongly suppressed the expression of cluster A genes in both WT and KO BATs and downregulated the expression of cluster B genes in KO BAT (Figure 6E, Supplementary Table S5). In the thermogenesis KEGG pathway, upregulated genes involved in mitochondrial oxidative phosphorylation were observed in WT (WT + AAV-GFP vs KO + AAV-GFP and WT + AAV-PRDM16 vs KO + AAV-PRDM16, Supplementary Figure 5A), and ectopic PRDM16 expression could promote their expression in both WT and KO BATs (WT + AAV-PRDM16 vs WT + AAV-GFP and KO + AAV-PRDM16 vs KO + AAV-GFP, Supplementary Figure 5B). However, KO BAT infected with AAV-PRDM16 still exhibited reduced expression of genes in mitochondrial oxidative phosphorylation compared to WT BAT infected with AAV-GFP (Supplementary Figure 5C). Taken together, abnormal weight gain in CPEB2-KO mice along with dysregulated muscle-related transcripts can be rectified by ectopic PRDM16 expression in BATs.

## 4 Discussion

Defective thermogenic and metabolic activities in the global CPEB2-KO mouse were primarily caused by the loss of CPEB2-activated translation in BAT. From previous and current studies, we identified that translation of both *Ucp1L* and *Prdm16* mRNAs depends on CPEB2. Moreover, the reduction in *Ucp1L* mRNA translation alone is insufficient to account for the elevated body weight gain in CPEB2-KO mice. Instead, the impaired *Prdm16* mRNA translation is the key cause, so ectopic expression of PRDM16 rescued gene expression to mitigate weight gain.

Despite the higher expression level of CPEB2 in the hypothalamus than BAT (Figure 2A), the obese phenotype in KO female mice led us to identify that CPEB2-activated translation of *Ucp1L* and *Prdm16* in BAT is critical for maintaining its thermogenic and metabolic activities. Insulin resistance is a key feature linking obesity to the metabolic syndrome, in which obesity and lipid accumulation are responsible for altered insulin action, thereby leading to glucose intolerance [44,45]. Because BAT transplantation improves whole body metabolism and glucose homeostasis [46,47] and CPEB2-KO mice have less active BAT, we examined systemic glucose tolerance (Supplementary Figure 6A) and insulin sensitivity (Supplementary Figure 6B) and found no significant differences between CPEB2-WT and -KO female mice at the age of ∼8–9 months. Histological analysis revealed no liver steatosis (i.e., fatty liver) in KO animals until they reached 12 mo old (Supplementary Figure 7A). Lipid metabolism and liver function in WT and obese KO female mice (∼8–9 mo old) were assessed by analyzing serum levels of triglycerides (TG), low- and high-density lipoprotein (LDL/HDL) and total cholesterol as well as glutamate oxaloacetate transaminase (GOT), glutamate pyruvate transaminase (GPT), and other biochemical factors. Only GPT activity was significantly increased, which likely resulted from progressive liver steatosis. Serum levels of thyroxine (T4) and leptin did not differ significantly though leptin levels showed a higher trend in obese KO mice due to their increased visceral WAT mass (Supplementary Figure 7B). Since insulin resistance often causes the enlargement of white adipocytes [48], the comparable size of lipid droplets and morphology of white adipocytes in WT and KO mice (Supplementary Figure 3) also supports the idea that dysregulated glucose homeostasis is unlikely the primary cause of obesity in the KO mice.

BAT is derived from *Myf5*-expressing myoblasts. Ectopic expression of PRDM16 in C2C12 cells and primary myoblasts promotes brown adipocyte differentiation depending on the co-expression of PPARγ. Conversely, knockdown of PRDM16 in preadipocytes isolated from BAT induces myogenic differentiation and gene expression *in vitro* [23]. However, mice with conditional ablation of *Prdm16* in *Myf5*-expressing cells (PRDM16-cKO^Myf5^) exhibit normal embryonic BAT development and limited changes in BAT-selective gene expression unless *Prdm3* is simultaneously ablated [49]. Notably, PRDM 16 is a key transcriptional regulator for the maintenance of BAT identity by promoting BAT-selective gene expression while suppressing WAT-specific gene expression [25,50], so PRDM16-cKO^Myf5^ mice show adult-onset loss of thermogenic activity and BAT dysfunction with increased expression of many WAT genes and whitening morphology in their iBAT at over 6 months of age [49]. Because the *Prdm3* transcript was not detected in our transcriptomes, we suspect this may explain why PRDM16 is essential for maintaining thermogenic and metabolic activity of BAT in adult mice. Similarly, PRDM16 also drives the browning of beige adipocytes in subcutaneous WAT, thereby improving metabolic phenotypes [24,51]. While PRDM16-cKO^Myf5^ mice exhibited smaller body size and did not become obese under a HFD [49], PRDM16-cKO^adipoQ^ mice, generated by crossing with *adiponectin*-Cre mice to ablate *Prdm16* in adipose tissues, developed overweight after 5-month of HFD feeding [51]. Although we cannot exclude the possibility that other mRNAs in BAT may be regulated by CPEB2, contributing to the obese phenotypes in CPEB2-KO mice, we believe another plausible explanation is that the loss of *Prdm16* in other Myf5-expressing lineage cells, such as certain cartilage progenitors in the ribs [52], may compromise overall health. This is supported by findings in *Prdm16*^+/−^ mice, which display abnormal cartilage and bone formation [53]. In our study, we observed increased body weight in CPEB2-KO and cKO^aP2^ mice at the age of ∼5–6 months, though decreased PRDM16 was observed in 2.5-mo-old CPEB2-deficient BAT (Figure 5D). From the transcriptomic comparison, genes showing a 2-fold upregulation are mostly related to muscle function. Many of these muscle genes are expressed at low levels in WT BAT but are elevated in KO BAT isolated from 5-mo-old mice (Figure 5A). RT-qPCR validation from different cohorts of BATs exhibited a wide range of fold changes (Figure 5E). In the BAT transcriptomes from the AAV rescue experiment (Figure 6D), we also observed that the expression of genes related to muscle function (cluster B) as a whole was elevated in KO BAT but could be downregulated by ectopic expression of flag-PRDM16 (Figure 6E). Notably, expression of flag-PRDM16 in both WT and KO BAT evidently suppressed immune response genes (cluster A). Our findings align with the previous study showing that PRDM16 inhibits the expression of interferon-stimulated genes by competing with the transcription factor, interferon regulatory factor 1 (IRF1), thereby repressing type I interferon response in both brown and white adipocytes to promote mitochondrial and thermogenic activity [54]. We found no changes in immune-related pathways in 4-5-mo-old CPEB2-KO BATs (Figure 5A and Supplementary Table 2); however, a significant increase was observed in KO BAT infected with AAV-GFP and collected at 26 weeks of age (Figure 6D–E). It is likely that increased fat mass (Figure 4D) in older CPEB2-KO mice contributed to the elevated inflammatory response [55].

PRDM16 is a master regulator that promotes adipose browning. PRDM16 haploinsufficiency shows a trend of increasing expression of muscle genes (i.e., *Myod*, *Mck* and *Myf6*) in iBAT and a reduced browning response in beige adipocytes stimulated by a β3AR agonist [23,24]. Mice lacking MyoD and half level of insulin-like growth factor 2 exhibit accelerated differentiation of brown preadipocytes and BAT hypertrophy, along with increased expression of PRDM16 and UCP1 [21]. Thus, the expression level of PRDM16 is crucial for promoting brown fat function. Posttranscriptional expression of PRDM16 could be negatively regulated by miR-133. Cold exposure markedly decreases miR-133 to promote PRDM16 expression and the browning response in BAT and beige adipocytes in subcutaneous WAT [22,56]. Another study used mice with the ablation of the m6A methyltransferase, *Mettl3*, in BAT and found that m6A modifications on PRDM16, PPARγ, and UCP1 transcripts are important for maintaining their expression for postnatal development of BAT and energy expenditure [57]. Because ectopic expression of CPEB2 and PRDM16 in the iBAT could suppress weight gain in CPEB2-KO mice, CPEB2-activated *Prdm16* translation is important to maintain the expression of this key regulator to uphold brown fat cell identity and function in adult mice.

From the available transcriptome data in peripheral blood mononuclear cells (PBMCs) and visceral adipose tissue of lean and obese subjects, no difference was observed in the mRNA levels of *CPEB2*, *UCP1* and *PRDM16* (Supplementary Figure 8A). However, the number of subjects was quite limited in these studies [[58], [59], [60]], and the expression of these transcripts in PBMCs is also very low. Interestingly, genome-wide association study (GWAS) data revealed an association between *CPEB2* and body weight in the Taiwan Biobank (*P* = 2 × 10^−6^, Supplementary Figure 8C), but not in the UK or Japan Biobanks. Moreover, no link between human obesity (elevated BMI) and any of the 3 genes (*CPEB2*, *UCP1* and *PRDM16*) was identified. Another GWAS revealed a significant association of the single nucleotide polymorphism, rs62409270, located at 4p15.32 in the *CPEB2* gene with low density lipoprotein cholesterol levels (*P* = 5.25 × 10^−9^) [61], suggesting that CPEB2-mediated translational control may have a role in regulating human body weight and lipid metabolism.

## Funding

This work was supported by National Health Research Institute (NHRI-EX109-10719SI) and National Science and Technology Council (NSTC, Taiwan [108-2320-B-001-020-MY3 and 111-2311-B-001-020-MY3] in Taiwan. W.-H.L. was supported by a postdoctoral fellowship from NSTC (2020-2021, 110-2811-B-001-569) and Academia Sinica (2022–2023).

## CRediT authorship contribution statement

**Wen-Hsin Lu:** Writing – original draft, Investigation, Formal analysis, Data curation. **Hui-Feng Chen:** Writing – original draft, Investigation, Formal analysis. **Pei-Chih King:** Methodology. **Chi Peng:** Formal analysis, Data curation. **Yi-Shuian Huang:** Writing – review & editing, Writing – original draft, Supervision, Funding acquisition, Conceptualization.

## Declaration of competing interest

The authors declare that they have no competing interests.

## Data Availability

Data will be made available on request.

## References

[1] Chen Z., Yang G., Zhou M., Smith M., Offer A., Ma J. (2006). Body mass index and mortality from ischaemic heart disease in a lean population: 10 year prospective study of 220,000 adult men. Int J Epidemiol.

[2] Mokdad A.H., Bowman B.A., Ford E.S., Vinicor F., Marks J.S., Koplan J.P. (2001). The continuing epidemics of obesity and diabetes in the United States. JAMA.

[3] Cannon B., Houstek J., Nedergaard J. (1998). Brown adipose tissue. More than an effector of thermogenesis?. Ann N Y Acad Sci.

[4] Chechi K., Carpentier A.C., Richard D. (2013). Understanding the brown adipocyte as a contributor to energy homeostasis. Trends Endocrinol Metabol.

[5] Cypess A.M., Lehman S., Williams G., Tal I., Rodman D., Goldfine A.B. (2009). Identification and importance of brown adipose tissue in adult humans. N Engl J Med.

[6] van Marken Lichtenbelt W.D., Vanhommerig J.W., Smulders N.M., Drossaerts J.M., Kemerink G.J., Bouvy N.D. (2009). Cold-activated brown adipose tissue in healthy men. N Engl J Med.

[7] Huang Y.S., Mendez R., Fernandez M., Richter J.D. (2023). CPEB and translational control by cytoplasmic polyadenylation: impact on synaptic plasticity, learning, and memory. Mol Psychiatr.

[8] Ivshina M., Lasko P., Richter J.D. (2014). Cytoplasmic polyadenylation element binding proteins in development, health, and disease. Annu Rev Cell Dev Biol.

[9] Lai Y.T., Chao H.W., Lai A.C., Lin S.H., Chang Y.J., Huang Y.S. (2020). CPEB2-activated PDGFRalpha mRNA translation contributes to myofibroblast proliferation and pulmonary alveologenesis. J Biomed Sci.

[10] Lai Y.T., Su C.K., Jiang S.T., Chang Y.J., Lai A.C., Huang Y.S. (2016). Deficiency of CPEB2-confined choline acetyltransferase expression in the dorsal motor nucleus of vagus causes hyperactivated parasympathetic signaling-associated bronchoconstriction. J Neurosci.

[11] Lu W.H., Yeh N.H., Huang Y.S. (2017). CPEB2 activates GRASP1 mRNA translation and promotes AMPA receptor surface expression, long-term potentiation, and memory. Cell Rep.

[12] Pascual R., Martin J., Salvador F., Reina O., Chanes V., Millanes-Romero A. (2020). The RNA binding protein CPEB2 regulates hormone sensing in mammary gland development and luminal breast cancer. Sci Adv.

[13] Chen H.F., Hsu C.M., Huang Y.S. (2018). CPEB2-dependent translation of long 3'-UTR Ucp1 mRNA promotes thermogenesis in brown adipose tissue. EMBO J.

[14] Lu W.H., Chang Y.M., Huang Y.S. (2020). Alternative polyadenylation and differential regulation of Ucp1: implications for Brown adipose tissue thermogenesis across species. Front Pediatr.

[15] Dai N., Zhao L., Wrighting D., Kramer D., Majithia A., Wang Y. (2015). IGF2BP2/IMP2-Deficient mice resist obesity through enhanced translation of Ucp1 mRNA and Other mRNAs encoding mitochondrial proteins. Cell Metabol.

[16] Kopecky J., Hodny Z., Rossmeisl M., Syrovy I., Kozak L.P. (1996). Reduction of dietary obesity in aP2-Ucp transgenic mice: physiology and adipose tissue distribution. Am J Physiol.

[17] Kozak L.P., Anunciado-Koza R. (2008). UCP1: its involvement and utility in obesity. Int J Obes.

[18] Takahashi A., Adachi S., Morita M., Tokumasu M., Natsume T., Suzuki T. (2015). Post-transcriptional stabilization of Ucp1 mRNA protects mice from diet-induced obesity. Cell Rep.

[19] Feldmann H.M., Golozoubova V., Cannon B., Nedergaard J. (2009). UCP1 ablation induces obesity and abolishes diet-induced thermogenesis in mice exempt from thermal stress by living at thermoneutrality. Cell Metabol.

[20] Zietak M., Kozak L.P. (2016). Bile acids induce uncoupling protein 1-dependent thermogenesis and stimulate energy expenditure at thermoneutrality in mice. Am J Physiol Endocrinol Metabol.

[21] Borensztein M., Viengchareun S., Montarras D., Journot L., Binart N., Lombes M. (2012). Double Myod and Igf2 inactivation promotes brown adipose tissue development by increasing Prdm16 expression. Faseb J : Official Publication of the Federation of American Societies for Experimental Biology.

[22] Yin H., Pasut A., Soleimani V.D., Bentzinger C.F., Antoun G., Thorn S. (2013). MicroRNA-133 controls brown adipose determination in skeletal muscle satellite cells by targeting Prdm16. Cell Metabol.

[23] Seale P., Bjork B., Yang W., Kajimura S., Chin S., Kuang S. (2008). PRDM16 controls a brown fat/skeletal muscle switch. Nature.

[24] Seale P., Conroe H.M., Estall J., Kajimura S., Frontini A., Ishibashi J. (2011). Prdm16 determines the thermogenic program of subcutaneous white adipose tissue in mice. J Clin Invest.

[25] Seale P., Kajimura S., Yang W., Chin S., Rohas L.M., Uldry M. (2007). Transcriptional control of brown fat determination by PRDM16. Cell Metabol.

[26] Enerback S., Jacobsson A., Simpson E.M., Guerra C., Yamashita H., Harper M.E. (1997). Mice lacking mitochondrial uncoupling protein are cold-sensitive but not obese. Nature.

[27] Giusti S.A., Vercelli C.A., Vogl A.M., Kolarz A.W., Pino N.S., Deussing J.M. (2014). Behavioral phenotyping of Nestin-Cre mice: implications for genetic mouse models of psychiatric disorders. J Psychiatr Res.

[28] He W., Barak Y., Hevener A., Olson P., Liao D., Le J. (2003). Adipose-specific peroxisome proliferator-activated receptor gamma knockout causes insulin resistance in fat and liver but not in muscle. Proc Natl Acad Sci USA.

[29] Tronche F., Kellendonk C., Kretz O., Gass P., Anlag K., Orban P.C. (1999). Disruption of the glucocorticoid receptor gene in the nervous system results in reduced anxiety. Nat Genet.

[30] Chen P.J., Huang Y.S. (2012). CPEB2-eEF2 interaction impedes HIF-1alpha RNA translation. EMBO J.

[31] Chen P.J., Weng J.Y., Hsu P.H., Shew J.Y., Huang Y.S., Lee W.H. (2015). NPGPx modulates CPEB2-controlled HIF-1alpha RNA translation in response to oxidative stress. Nucleic Acids Res.

[32] Whittle A.J., Carobbio S., Martins L., Slawik M., Hondares E., Vazquez M.J. (2012). BMP8B increases brown adipose tissue thermogenesis through both central and peripheral actions. Cell.

[33] Viegas M.S., Martins T.C., Seco F., do Carmo A. (2007). An improved and cost-effective methodology for the reduction of autofluorescence in direct immunofluorescence studies on formalin-fixed paraffin-embedded tissues. Eur J Histochem.

[34] Chen C.C., Sun C.P., Ma H.I., Fang C.C., Wu P.Y., Xiao X. (2009). Comparative study of anti-hepatitis B virus RNA interference by double-stranded adeno-associated virus serotypes 7, 8, and 9. Mol Ther.

[35] Clark R.G., Tarttelin M.F. (1982). Some effects of ovariectomy and estrogen replacement on body composition in the rat. Physiol Behav.

[36] Gale S.K., Van Itallie T.B. (1979). Genetic obestiy: estrogenic influences on the body weight and food intake of lean and obese adult Zucker (fa/fa) rats. Physiol Behav.

[37] Kontani Y., Wang Y., Kimura K., Inokuma K.I., Saito M., Suzuki-Miura T. (2005). UCP1 deficiency increases susceptibility to diet-induced obesity with age. Aging Cell.

[38] Lowell B.B., Spiegelman B.M. (2000). Towards a molecular understanding of adaptive thermogenesis. Nature.

[39] Morrison S.F., Madden C.J., Tupone D. (2014). Central neural regulation of brown adipose tissue thermogenesis and energy expenditure. Cell Metabol.

[40] Ge S.X., Son E.W., Yao R. (2018). iDEP: an integrated web application for differential expression and pathway analysis of RNA-Seq data. BMC Bioinf.

[41] Ge X. (2021). iDEP web application for RNA-seq data analysis. Methods Mol Biol.

[42] Sherman B.T., Hao M., Qiu J., Jiao X., Baseler M.W., Lane H.C. (2022). DAVID: a web server for functional enrichment analysis and functional annotation of gene lists (2021 update). Nucleic Acids Res.

[43] Wang W., Ishibashi J., Trefely S., Shao M., Cowan A.J., Sakers A. (2019). A PRDM16-driven metabolic signal from adipocytes regulates precursor cell fate. Cell Metabol.

[44] Bagry H.S., Raghavendran S., Carli F. (2008). Metabolic syndrome and insulin resistance: perioperative considerations. Anesthesiology.

[45] Morigny P., Houssier M., Mouisel E., Langin D. (2016). Adipocyte lipolysis and insulin resistance. Biochimie.

[46] Gunawardana S.C., Piston D.W. (2012). Reversal of type 1 diabetes in mice by brown adipose tissue transplant. Diabetes.

[47] Stanford K.I., Middelbeek R.J., Townsend K.L., An D., Nygaard E.B., Hitchcox K.M. (2013). Brown adipose tissue regulates glucose homeostasis and insulin sensitivity. J Clin Invest.

[48] Guilherme A., Virbasius J.V., Puri V., Czech M.P. (2008). Adipocyte dysfunctions linking obesity to insulin resistance and type 2 diabetes. Nat Rev Mol Cell Biol.

[49] Harms M.J., Ishibashi J., Wang W., Lim H.W., Goyama S., Sato T. (2014). Prdm16 is required for the maintenance of brown adipocyte identity and function in adult mice. Cell Metabol.

[50] Kajimura S., Seale P., Tomaru T., Erdjument-Bromage H., Cooper M.P., Ruas J.L. (2008). Regulation of the brown and white fat gene programs through a PRDM16/CtBP transcriptional complex. Genes Dev.

[51] Cohen P., Levy J.D., Zhang Y., Frontini A., Kolodin D.P., Svensson K.J. (2014). Ablation of PRDM16 and beige adipose causes metabolic dysfunction and a subcutaneous to visceral fat switch. Cell.

[52] Haldar M., Hancock J.D., Coffin C.M., Lessnick S.L., Capecchi M.R. (2007). A conditional mouse model of synovial sarcoma: insights into a myogenic origin. Cancer Cell.

[53] Kaneda-Nakashima K., Igawa K., Suwanruengsri M., Naoyuki F., Ichikawa T., Funamoto T. (2022). Role of Mel1/Prdm16 in bone differentiation and morphology. Exp Cell Res.

[54] Kissig M., Ishibashi J., Harms M.J., Lim H.W., Stine R.R., Won K.J. (2017). PRDM16 represses the type I interferon response in adipocytes to promote mitochondrial and thermogenic programing. EMBO J.

[55] Aruwa C.E., Sabiu S. (2024). Adipose tissue inflammation linked to obesity: a review of current understanding, therapies and relevance of phyto-therapeutics. Heliyon.

[56] Trajkovski M., Ahmed K., Esau C.C., Stoffel M. (2012). MyomiR-133 regulates brown fat differentiation through Prdm16. Nat Cell Biol.

[57] Wang Y., Gao M., Zhu F., Li X., Yang Y., Yan Q. (2020). METTL3 is essential for postnatal development of brown adipose tissue and energy expenditure in mice. Nat Commun.

[58] Chen S., Song P., Wang Y., Wang Z., Xue J., Jiang Y. (2023). CircMAPK9 promotes adipogenesis through modulating hsa-miR-1322/FTO axis in obesity. iScience.

[59] Hulsmans M., Geeraert B., De Keyzer D., Mertens A., Lannoo M., Vanaudenaerde B. (2012). Interleukin-1 receptor-associated kinase-3 is a key inhibitor of inflammation in obesity and metabolic syndrome. PLoS One.

[60] Jung U.J., Seo Y.R., Ryu R., Choi M.S. (2016). Differences in metabolic biomarkers in the blood and gene expression profiles of peripheral blood mononuclear cells among normal weight, mildly obese and moderately obese subjects. Br J Nutr.

[61] Graham S.E., Clarke S.L., Wu K.H., Kanoni S., Zajac G.J.M., Ramdas S. (2021). The power of genetic diversity in genome-wide association studies of lipids. Nature.

