# Supplementary material for: CPEB2-activated Prdm16 translation promotes brown adipocyte function and prevents obesity
Source: Mol Metab. 2024 Sep 19;89:102034. doi: 10.1016/j.molmet.2024.102034 (PMC11462068; doi:10.1016/j.molmet.2024.102034)
Supplement: Multimedia component 1 [file mmc1.pdf]

CPEB2-actvated *Prdm16* translation promotes brown adipocyte function and prevents obesity

Wen-Hsin Lu, Hui-Feng Chen, Pei-Chih King, Chi Peng
and Yi-Shuian Huang

Supplementary Methods
Supplementary Table 1
Supplementary Figures 1-8

Supplementary Methods

Cervical smear test and the plasma levels of estradiol and progesterone

Vaginal smears were performed daily between 10:00 and 11:00 AM for 16 consecutive days. The vagina was flushed with 10 μl saline to suspend epithelial and immune cells. The retrieved fluid was then placed onto a glass slide and allowed to dry before staining with Liu’ s Stain A and B. Microscopic evaluation of cell morphology was performed to determine the stages of the estrous cycle. Following the assessment of the estrous cycle stages, plasma samples from female mice were collected. The levels of estradiol and progesterone were quantified using an enzyme-linked immunosorbent assay (ELISA) kit (Cayman Chemical, USA), according to the manufacturer's instructions.

Oral glucose tolerance test (OGTT), insulin tolerance test (ITT) and serum biochemistry

The 10-mo-old female mice were used for the assays. For OGTT, mice were fasted overnight and orally dosed with 1.5 g/kg glucose. For ITT, mice were fasted for 4 h prior to intraperitoneal injection of 0.75 U/kg of insulin. Blood glucose values at the designated time points after glucose or insulin administration were determined by Accu-Chek glucometer (Roche). The serum of experimental animals was obtained after overnight fasting. Serum biochemical measurements were performed by Taiwan Mouse Clinic staffs using the following kits according to the manufacturer's instructions: LDL, HDL and Cholesterol were measured using HDL and LDL/VLDL Quantification Colorimetric Assay Kit (BioVision, CA, USA). Thyroxine (T4) was measured using an enzyme-linked immunosorbent assay kit (CALBIOTECH, CA, USA). Leptin was determined by Mouse/Rat Leptin Quantikine ELISA Kit (R&D Systems, Inc. Minneapolis, USA).

Supplementary Table 1 Primer sequences for qPCR

**qPCR**	**Forward**	**Reverse**
β3AR	CAGCCAGCCCTGTTGAAG	GAAGATGGGGATCAAGCAAG
Ucp1	GGCCTCTACGACTCAGTCCA	TAAGCCGGCTGAGATCTTGT
Dio2	CTGCGCTGTGTCTGGAAC	GGAGCATCTTCACCCAGTTT
Ampk	CCTTCGGGAAAGTGAAGGT	GAATCTTCTGCCGGTTGAGT
Pparγ	AAGACAACGGACAAATCACCA	GGGGGTGATATGTTTGAACTTG
Srebp1	ACAAGATTGTGGAGCTCAAAGAC	GCGCAAGACAGCAGATTTATT
Hsl	AGCGCTGGAGGAGTG TTTT	CCGCTCTCCAGTTGAACC
Acta1	CTATTCCTTCGTGACCACAGC	CGGTGGCCATCTCATTCT
Myh1	CAATGGCTGCATCAACTATGA	GCCATAGGTGTTCTTGAACTGG
Myl1	AATCAAAGGTCAAGGCCTACAA	GAATTTGGCCAGGTTGACAT
Prdm16	CCTAAGGTGTGCCCAGCA	CACCTTCCGCTTTTCTACCC
Gapdh	GCCAAAAGGGTCATCATCTC	CACACCCATCACAAACATGG

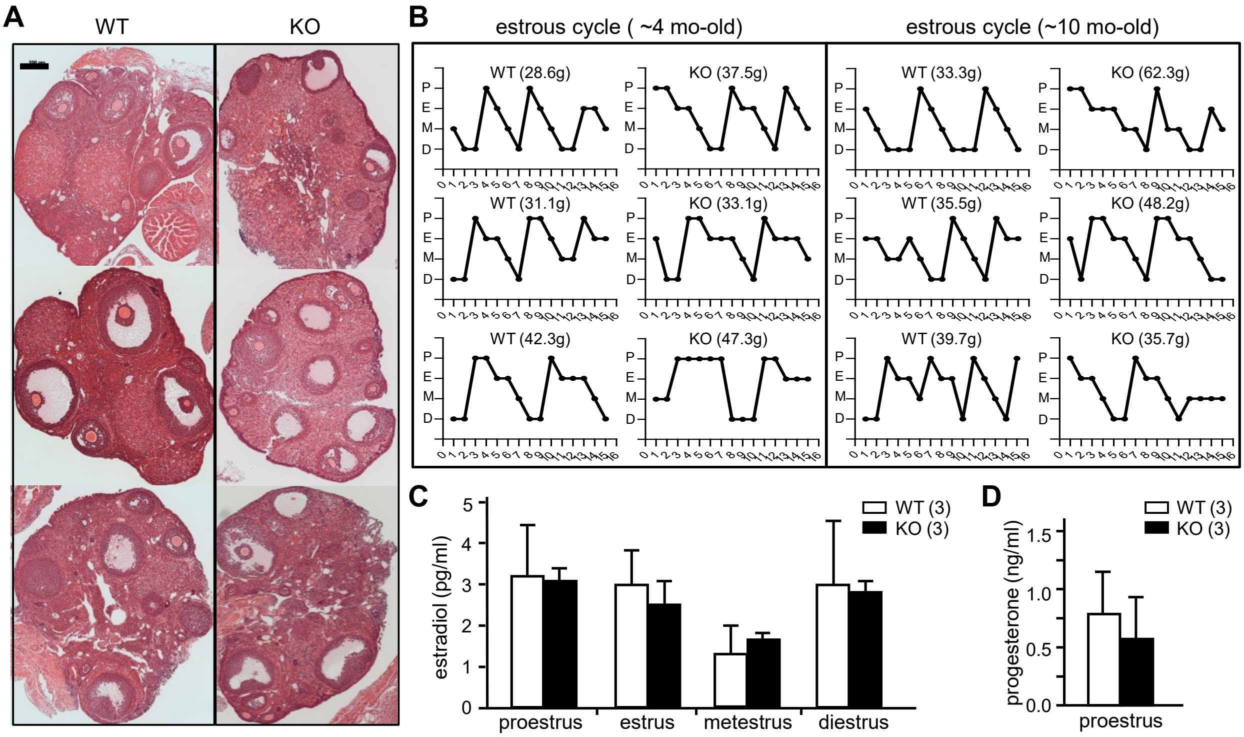

Supplementary Figure 1: CPEB2-KO females have normal levels of estrogen and progesterone.

(A) Hematoxylin and eosin-stained sections of ovaries from 10-mo-old WT and KO mice (n = 3 mice per group). Scale, 200 µm. **(B)** The estrous cycles of 4- and 10-mo-old female WT and KO mice. Body weights are in parentheses. P, proestrus; E, estrus; M, metestrus; D, diestrus. **(C)** The serum levels of estradiol and **(D)** progesterone in WT and KO females (n = 3 mice per group).

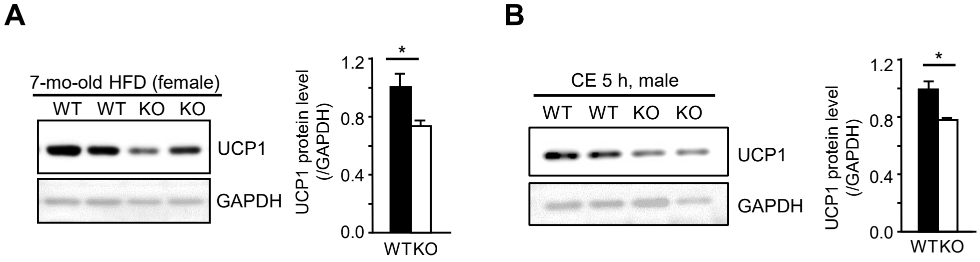

Supplementary Figure 2: Reduced UCP1 expression in CPEB2-deficient BAT under a HFD or cold exposure.

The BAT samples collected from **(A)** female mice under a HFD for 5 months (n =3 per group) and **(B)** male mice after 5 h cold exposure (CE, n = 3 per group) were used for Western blotting analysis. The quantified results normalized with GAPDH are mean ± SEM. **P* < 0.05, Student's *t* test.

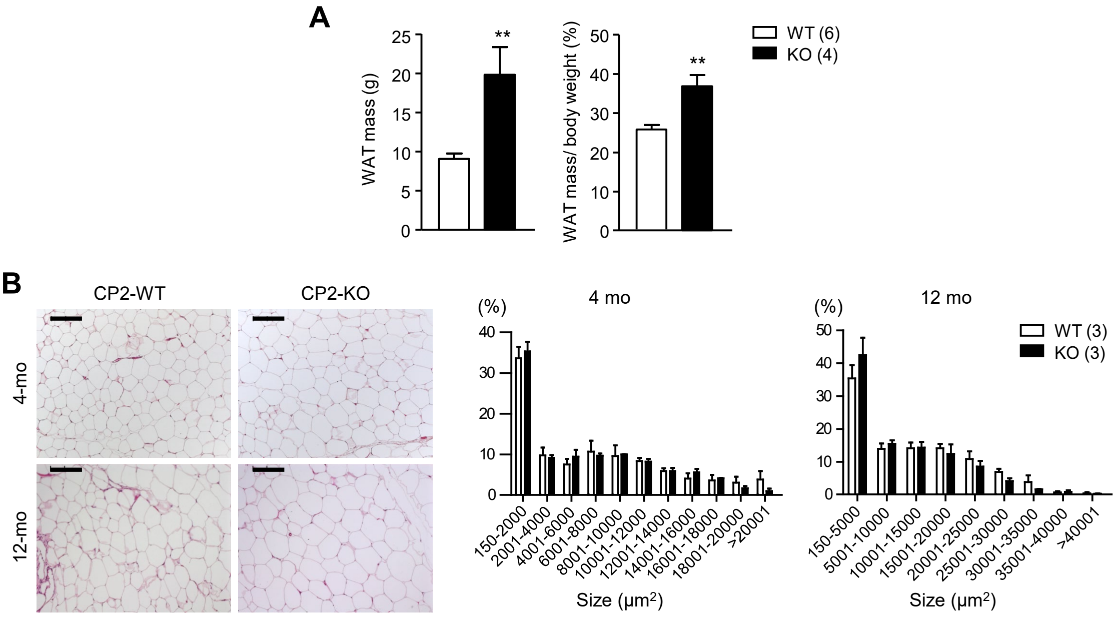

Supplementary Figure 3: CPEB2-KO female mice show increased white adipose tissue (WAT) mass but comparable adipocyte size to WT littermates

(A) Weight of visceral WAT and body fat percentage (WAT weight/body weight) in 8-9-mo-old CPEB2-WT and -KO female mice (n = 6-4). $^{**}P < 0.01$ by Student's *t* test. **(B)** Hematoxylin and eosin sections of visceral WAT from WT and CPEB2-KO female mice at 4 and 12 mo old. Scale bars, 1 mm. Adipocyte size distribution is comparable between the 2 groups of mice. Three sections per mouse and 3 mice per group were analyzed.

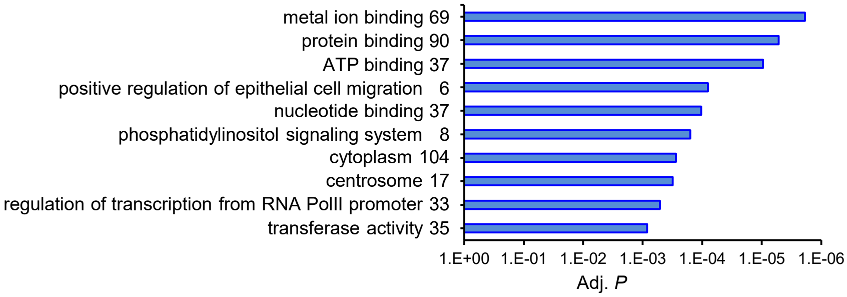

Supplementary Figure 4: GO categories of downregulated genes in CPEB2-KO BAT.
Gene ontology (GO) analysis of 240 differentially expressed genes (DEGs) that are downregulated ($\geq$ 2-fold change and an FDR $\leq$ 0.5) in CPEB2-KO BAT.

A

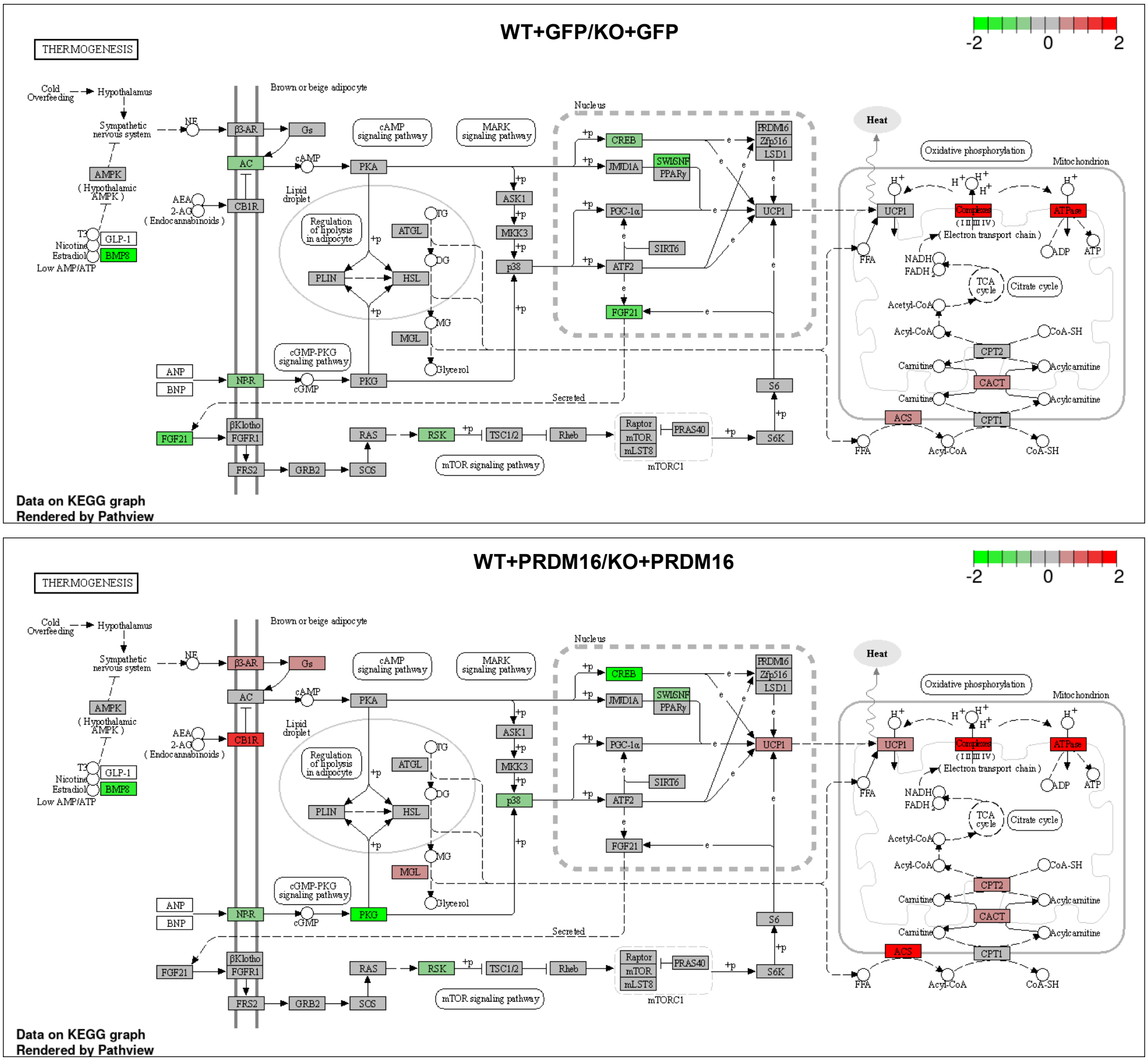

Supplementary Figure 5: Differentially expressed genes (DEGs) in the 'Thermogenesis' KEGG Pathway. AAVs expressing flag-PRDM16 or GFP were delivered bilaterally to iBATs of CPEB2-WT and -KO mice (n = 3 mice per group). After the completion of body weight measurements, iBAT samples were collected for RNA-seq and transcriptomic profiling. **(A-C)** DEGs between the designated 2 groups (WT+GFP, WT+PRDM16, KO+GFP, KO+PRDM16) are presented in various shades of red (upregulated) and green (downregulated) .

B

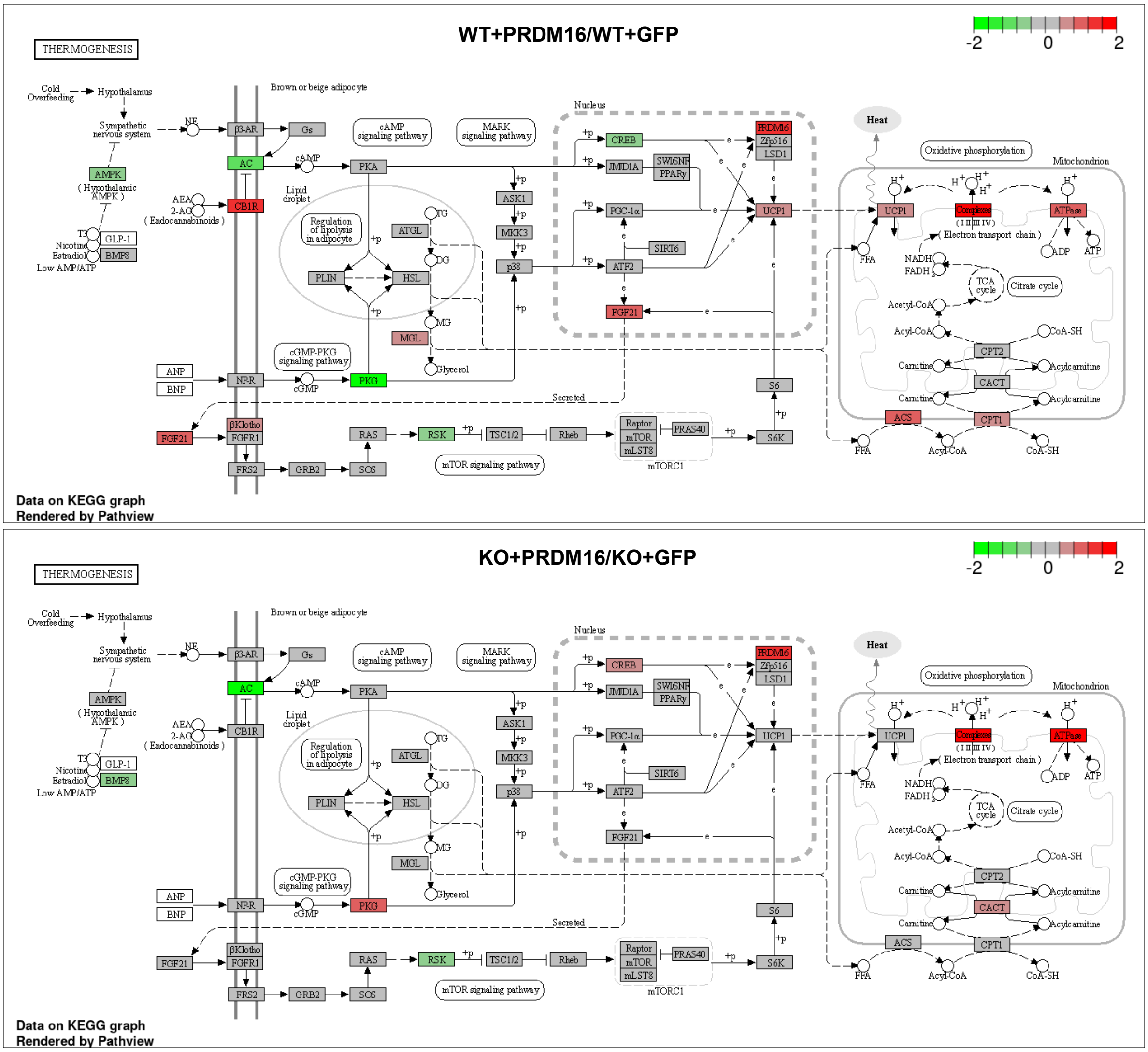

C

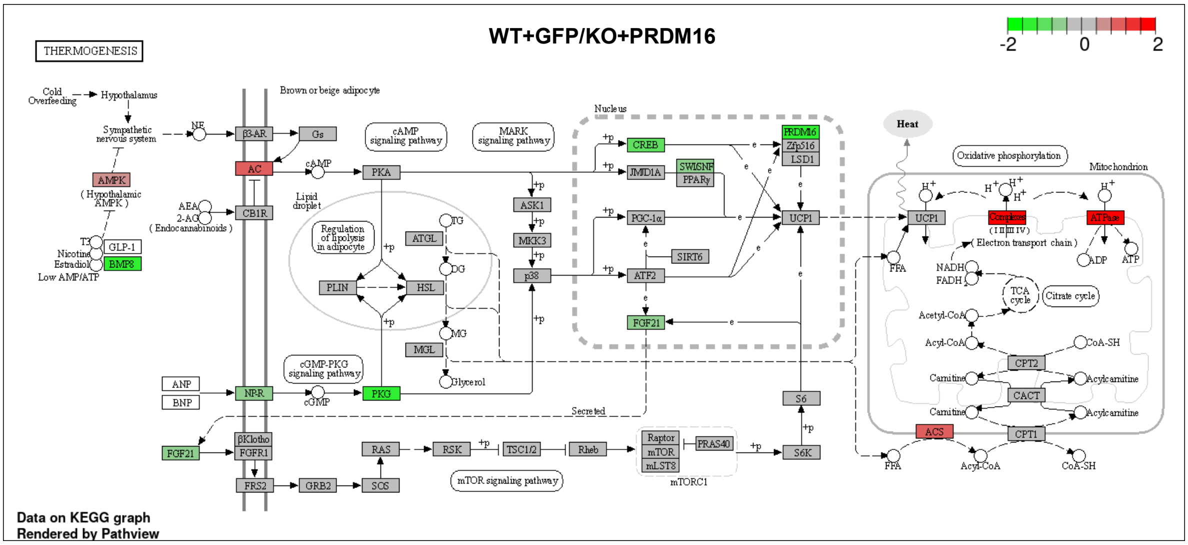

Supplementary Figure 5 continued: DEGs in the ‘Thermogenesis’ KEGG Pathway.

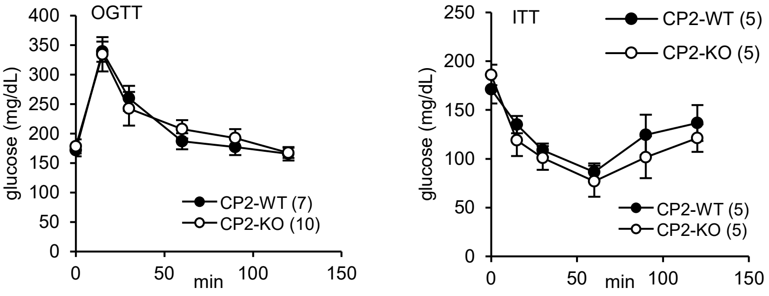

Supplementary Figure 6: Normal systemic glucose and insulin tolerance responses in CPEB2 KO female mice. (A) Oral glucose tolerance test (OGTT) in 9-10-mo-old female mice. **(B)** Insulin tolerance test (ITT) in 10-mo-old female mice. Numbers in parentheses are number of mice in each group.

A

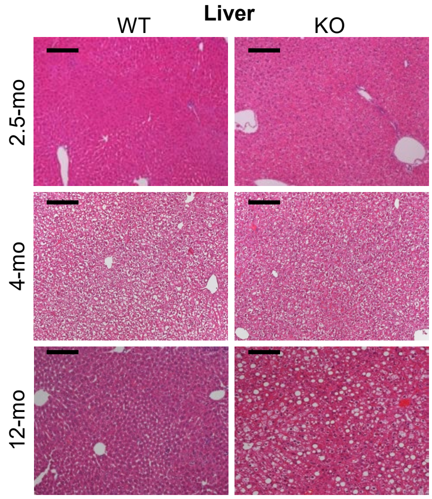

B

ITEM	Units	WT	KO
TG	mg/dl	96.6 ± 16.5 (5)	85.2 ± 8.5 (6)
LDL	mg/dL	16.29 ± 2.52 (8)	19.56 ± 3.44 (8)
HDL	mg/dL	104.4 ± 7.65 (8)	113.4 ± 13.56 (8)
Cholesterol	mg/dL	127.7 ± 10.53 (8)	129.2 ± 10.76 (8)
GOT	U/l	128.5 ± 31.51 (10)	176.4 ± 31.06 (10)
***GPT**	**U/l**	**54.60 ± 12.73 (10)**	**107.5 ± 19.86 (10)**
LDH	U/l	315.0 ± 50.24 (6)	351.8 ± 90.18 (5)
ALP	U/l	220.0 ± 29.30 (6)	281.4 ± 58.19 (5)
TBIL	mg/dl	0.73 ± 0.05 (6)	0.6 ± 0.0 (5)
TP	g/dl	5.32 ± 0.12(6)	5.24 ± 0.46 (5)
ALB	g/dl	2.87 ± 0.13 (6)	2.64 ± 0.17 (5)
IP	mg/dl	8.10 ± 0.51 (6)	9.02 ± 0.61 (5)
T4	μg/dL	4.96 ± 0.53 (8)	4.88 ± 0.52 (8)
Leptin	pg/ml	2018±403.7 (8)	3261±760 (8)

Supplementary Figure 7: Hepatic morphology and serum biochemical measurements in CPEB2-WT and -KO mice. (A) Hematoxylin and eosin sections of liver samples from WT and KO mice of different ages. Scale bars, 1 mm. **(B)** Serum biochemistry analysis. TG, triglycerol; LDH, low density lipoprotein; HDL, high density lipoprotein; GOT, glutamate oxaloacetate transaminase; GPT, glutamate pyruvate transaminase; LDH, lactate dehydrogenase; ALP, alkaline phosphatase; TBIL, total bilirubin; TP, total protein; ALB, albumin; IP, inorganic phosphate; T4, thyroxine. Numbers in parentheses denote the number of mice were used to collect serum samples for the study. *$P < 0.05$, Student's *t* test.

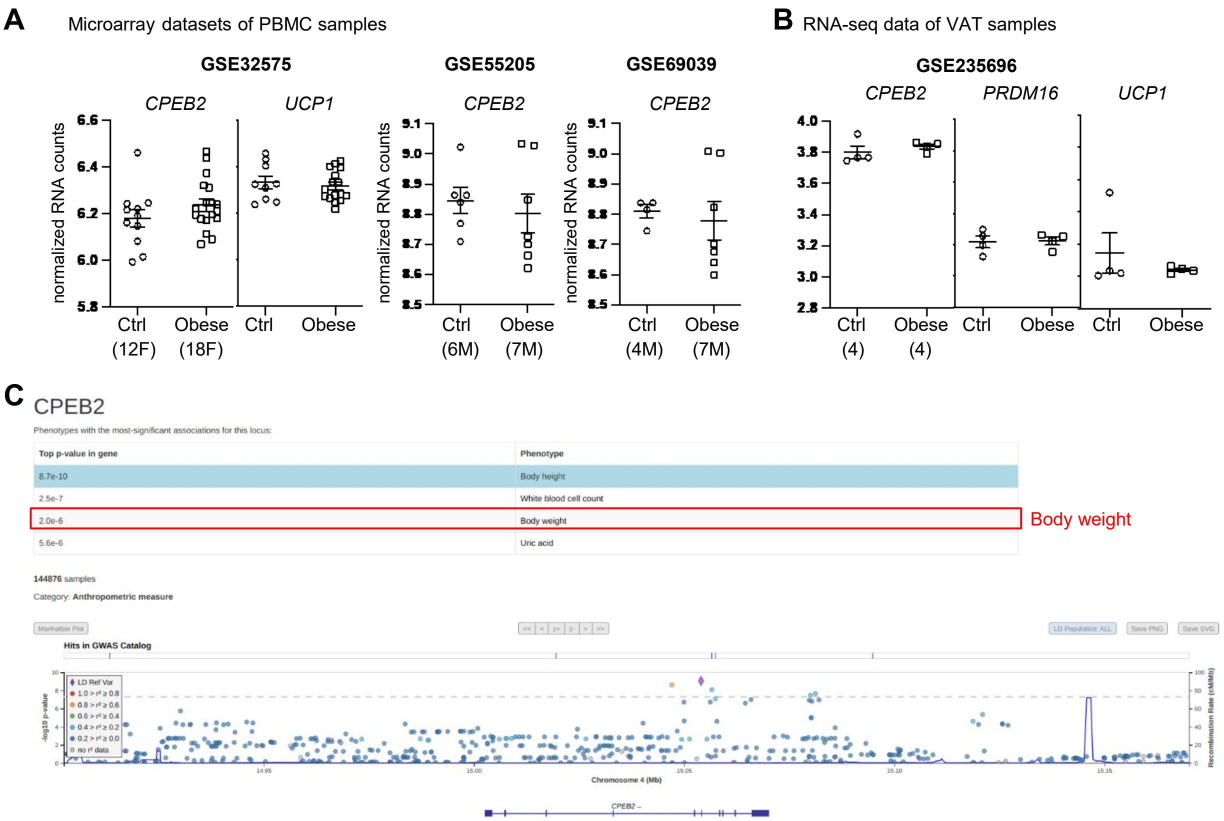

Supplementary Figure 8: Association of the *CPEB2* gene with body height and weight in the Taiwan Biobank. (A) The mRNA levels in peripheral blood mononuclear cells (PBMCs). GSE32575: The body mass index (BMI) for age-matched control and obese women is 20.3 ± 0.5 and 45.1 ± 1.4 kg/m^2, respectively. GSE55205: The BMI range for control and obese men is 18.5-22.9 and ≥ 30 kg/m^2, respectively. GSE69039: The BMI range for control and obese men is 18.5-23 and 27.5-30 kg/m^2, respectively. Numbers in parentheses are the number of subjects (F: female or M: male) in each group. **(B)** The mRNA levels in visceral adipose tissue (VAT). The BMI range for control and obese subjects (gender unspecified) is 18.5-23.9 and ≥ 28 kg/m^2, respectively. **(C)** GWAS data from the Taiwan Biobank show an association between the *CPEB2* gene and both body height (P = 8.7 x10^{-10}) and weight (P = 2 x10^{-6}). No association was identified between *UCP1* or *PRDM16* and body weight or obesity.
